# Amplification of spatially isolated adenosine pathway by tumor–macrophage interaction induces anti-PD1 resistance in hepatocellular carcinoma

**DOI:** 10.1186/s13045-021-01207-x

**Published:** 2021-11-27

**Authors:** Jia-Cheng Lu, Peng-Fei Zhang, Xiao-Yong Huang, Xiao-Jun Guo, Chao Gao, Hai-Ying Zeng, Yi-Min Zheng, Si-Wei Wang, Jia-Bin Cai, Qi-Man Sun, Ying-Hong Shi, Jian Zhou, Ai-Wu Ke, Guo-Ming Shi, Jia Fan

**Affiliations:** 1 Department of Liver Surgery and Transplantation, Zhongshan Hospital, Fudan University, Shanghai, 200032 China; 2 Liver Cancer Institute, Fudan University, Shanghai, 200032 China; 3 Key Laboratory of Carcinogenesis and Cancer Invasion, Ministry of Education of the People’s Republic of China, Shanghai, 200032 China; 4 Institutes of Biomedical Sciences, Fudan University, Shanghai, 200031 China; 5 Department of Pathology, Zhongshan Hospital, Fudan University, Shanghai, 200032 China

**Keywords:** Hepatocellular carcinoma, Exosomal circRNA, Macrophage, CD39, ATP–adenosine pathway

## Abstract

**Background:**

Immune checkpoint blockade resistance narrows the efficacy of cancer immunotherapies, but the underlying mechanism remains elusive. Delineating the inherent mechanisms of anti-PD1 resistance is important to improve outcome of patients with advanced HCC.

**Method:**

The level of cricTMEM181 was measured in HCC patients with anti-PD1 therapy by RNA sequencing and then confirmed by qPCR and Sanger sequencing. Immune status in tumor microenvironment of HCC patients or mice models was evaluated by flow cytometry and IHC. Exosomes from HCC cell lines were isolated by ultracentrifugation, and their internalization by macrophage was confirmed by immunofluorescence. The underlying mechanism of HCC-derived exosomal circTMEM181 to macrophage was confirmed by SILAC, RNA FISH and RNA immunoprecipitation. The ATP–ADO pathway amplified by HCC–macrophage interaction was evaluated through ATP, AMP and ADO measurement and macrophage-specific CD39 knockout mice. The role of circTMEM181 in anti-PD1 therapy and its clinical significance were also determined in our retrospective HCC cohorts.

**Results:**

Here, we found that circTMEM181 was elevated in hepatocellular carcinoma (HCC) patients responding poorly to anti-PD1 therapy and in HCC patients with a poor prognosis after operation. Moreover, we also found that high exosomal circTMEM181 favored the immunosuppressive microenvironment and endowed anti-PD1 resistance in HCC. Mechanistically, exosomal circTMEM181 sponged miR-488-3p and upregulated CD39 expression in macrophages. Using macrophage-specific CD39 knockout mice and pharmacologic approaches, we revealed a novel mode of anti-PD1 resistance in HCC. We discovered that cell-specific CD39 expression in macrophages and CD73 expression in HCC cells synergistically activated the eATP–adenosine pathway and produced more adenosine, thereby impairing CD8^+^ T cell function and driving anti-PD1 resistance.

**Conclusion:**

In summary, HCC-derived exosomal circTMEM181 contributes to immunosuppression and anti-PD1 resistance by elevating CD39 expression, and inhibiting the ATP–adenosine pathway by targeting CD39 on macrophages can rescue anti-PD1 therapy resistance in HCC.

**Graphical Abstract:**

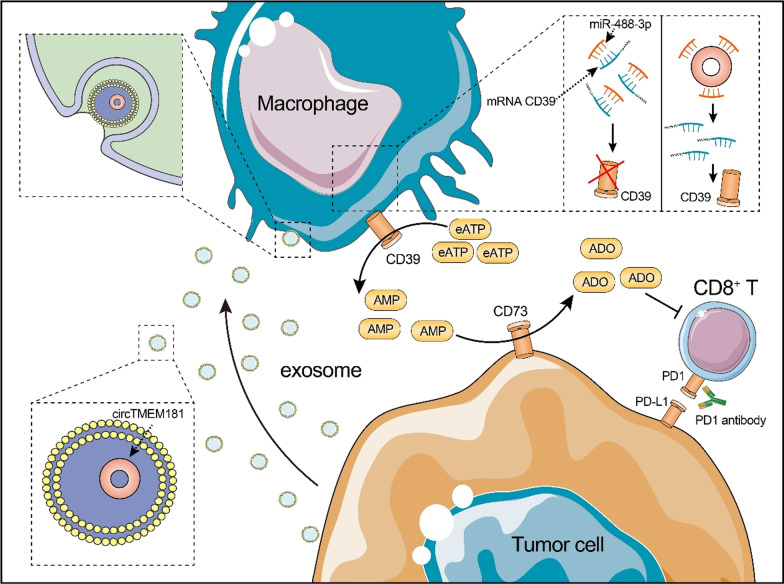

**Supplementary Information:**

The online version contains supplementary material available at 10.1186/s13045-021-01207-x.

## Background

Hepatocellular carcinoma (HCC) is the third most common cause of cancer-related deaths worldwide but lacks effective therapy because most patients are not diagnosed until they reach an advanced stage[1, 2]. Treatment with immunotherapy, particularly immune checkpoint blockade, can lead to benefit in the clinical setting [3]. For example, anti-PD1 antibody, which blocks the programmed cell death 1 protein (PD1)/programmed cell death 1 ligand 1 (PD-L1) axis to inhibit effector immune cell exhaustion, has revolutionized treatment of multiple advanced malignancies [3–5]. Despite this great success, many preclinical and clinical studies suggest that congenital and acquired resistance to anti-PD1 treatment frequently occurs, resulting in tumor relapse and treatment failure in HCC patients [6]. Therefore, delineating the inherent mechanisms of anti-PD1 resistance is important to improve outcome of patients with advanced HCC.

T cell exhaustion in the tumor microenvironment (TME) has emerged as a key mechanism for anti-PD1/PD-L1 resistance in malignancies, including HCC. For example, multifarious immunomodulatory receptors and cytokines in the TME contribute to the exhausted CD8^+^ T cell phenotype in HCC [7]. Aberrant accumulation of tumor-associated macrophages (TAMs) also creates an immunosuppressive TME and facilitates immune escape [8]. In addition, activation of adenosine signaling is an importantly immunosuppressive feature of HCC, characterized by a high level of CD73 and CD39 in the TME [9, 10]. As the end-product regularly catalyzed by CD39 and CD73 in metabolic pathways, adenosine is an important immunosuppressive factor that promotes an exhausted phenotype in natural killer (NK) and T cells in the TME [11, 12]. However, the activation mechanism of adenosine signaling and its role in the immunosuppressive microenvironment and immunotherapy for HCC need to be further evaluated.

As the products of back-splicing events, circular RNAs (circRNAs) belong to a class of noncoding RNAs. The ring structure of circRNAs prevents them from degradation and leads to their high stability [13]. Recently, circRNAs were identified as important mediators during many biological processes, mainly through sponging miRNAs or facilitating the function of signal proteins [14]. Importantly, aberrant circRNA accumulation occurs in various types of tumors [15]. For example, expression of several circRNAs correlates with disease progression of prostate cancer, lung cancer, and HCC, and circFECR1 promotes breast cancer cell metastasis by regulating DNA methylating and demethylating enzymes [16–18]. Furthermore, recent studies demonstrated that circRNA dysregulation promotes resistance to immunotherapy in HCC [19]. These findings indicate that circRNAs participate in the process of tumor development in multiple dimensions. Particularly, the interactions between tumor and immune cells are affected by circRNAs [19]. For example, our previous study demonstrated that HCC-derived exosomal cricUHRF1 impairs the function of NK cells to confer anti-PD1 resistance in HCC [20]. Therefore, circRNAs could extend the current theory of cancer immunotherapy resistance and require further study.

Here, we found increased circTMEM181 expression in puncture biopsies of tumor tissues from anti-PD1 antibody-resistant HCC patients (tumor response showed disease progression) compared to those from anti-PD1-sensitive patients (partial response). By in situ hybridization, we found that circTMEM181 was upregulated in most HCC tissues compared to the corresponding para-tumor tissues, and elevated circTMEM181 was related to short overall survival (OS) and high recurrence rates in HCC patients after operation. Mechanistically, we uncovered that HCC cells could upregulate CD39 expression in macrophages by exosomal circTMEM181. Importantly, HCC cells only expressed CD73, and macrophages only expressed CD39, jointly achieving full-blown activation of the adenosine pathway in the HCC microenvironment and inducing CD8^+^ T exhaustion and PD1 antibody resistance. Moreover, transgenic mouse experiments also showed activation of the adenosine pathway and anti-PD1 resistance in a CD39-dependent manner. Thus, our results reveal that circTMEM181 serves as a promoter in HCC progression and anti-PD1 therapy resistance and characterize the adenosine pathway activation in HCC.

## Methods

### Tissue microarray, human samples, and cell lines

Tumor tissue samples and/or adjacent nontumorous samples were obtained from HCC patients who underwent curative liver resection between 2018 and 2019 at the Liver Cancer Institute, Zhongshan Hospital, Fudan University. Biopsy samples and blood samples were collected from advanced HCC patients before nivolumab therapy. Tissue microarray (TMA) was constructed by Shanghai Biochip Co. Ltd. (Shanghai, China). All the HCC tissues were reviewed and confirmed histologically by H&E staining. Detailed materials are listed in Additional file 2. The collection of human samples was approved by the Zhongshan Hospital Research Ethics Committee. Written informed consent was obtained from each patient.

All the identified cell lines were provided from Key Laboratory of Carcinogenesis and Cancer Invasion, Ministry of Education of P.R.C or National Collection of Authenticated Cell Cultures, P.R.C. Cell lines HepG2, HCCLM3, MHC97H, Huh-7, PLC/PRF/5, H22, and Hepa1-6 were cultured in DMEM with 10% fetal bovine serum. The cell lines THP-1 and Li-7 were cultured in RPMI-1640 with 10% fetal bovine serum. All the cells were cultured at 37 °C in a 5% CO_2_ incubator.

### qPCR, Western blot analysis, and ELISA

qPCR and Western blot analysis were performed as our previous study described [20]. The antibodies, primers, and detailed materials are listed in Additional file 2.

### RNA ISH, RNA FISH, RNA immunoprecipitation, and RNA sequencing

RNA ISH, RNA FISH, RNA pulldown, and RNA sequencing were performed as our previous study described [19, 20]. The detailed methods and materials are listed in Additional file 2.

### Mice model, in vivo tumor models, and optical in vivo imaging

Male C57BL/6 J mice aged 6 weeks were purchased from Vital River Laboratory Animal Co., Ltd. (Beijing, China). C57BL/6-Entpd1^fl/fl^ was constructed by Cyagen (Suzhou, Jiangsu, China). C57BL/6-Lyz2^CreERT2^ were constructed by Shanghai Model Organisms Center, Inc. (Shanghai, China). C57BL/6-Lyz2^CreERT2^ × C57BL/6-Entpd1^fl/fl^ mice were constructed in our laboratory. Silencing ENTPD1 in macrophage was activated by treating with tamoxifen (dissolved in corn oil, 20 mg/ml by shaking overnight at 37 °C) i.p. (100ul/mouse, for 5 days).

Subcutaneous tumor xenograft in mice model: 1 × 10^6^ H22 mice liver tumor cells in 0.1 ml of DMEM were implanted subcutaneously in the right flank of the mice. The tumor volume was screened by caliper every 3 days.

Orthotopic tumor xenograft in mice model: 1 × 10^6^ H22 mice liver tumor cells labeled luciferase (H22-luc) in 50uL Matrigel were implanted to construct orthotopic liver xenografts in the mouse. The tumors were screened by optical in vivo Imaging system.

Treatment was performed 7 days later after tumor construction. Anti-mouse PD1 (HRP00262-012, 0.85% saline vehicle, provided as gifts by Hengrui Medicine Com., Jiangsu, China) was intraperitoneally injected (10 mg/kg, 3 times every week, for 2 weeks). POM1 was intraperitoneally injected (5 mg/kg, 4 times every week, for 2 weeks). Clophosome was intraperitoneally injected (0.2 μl/mouse, every week, for 2 weeks).

Optical in vivo imaging is detailed in Additional file 2.

### Exosome isolation and tumor dissociation

In brief, exosomes were pelleted by ultracentrifugation and used for the following experiments. Tumor dissociation was performed following the protocol from Tumor Dissociation Kit, mouse (#130–096-730, Miltenyi Biotec) or Tumor Dissociation Kit, human (#130–095-929, Miltenyi Biotec). All the detailed methods are listed in Additional file 2.

### Flow cytometry and sorting

Isolated single-cell suspensions from the blood or tissue of human or mouse were centrifuged (350 g, 5 min, at 4 °C) in a centrifuge and resuspended in staining buffer (1% FBS in PBS) on ice. Fc Blocks (1 μl for each tube) were used before staining.

For surface staining, the antibodies were incubated with cell suspensions for 20 min in dark place at 4 °C. For intracellular staining, saponin was used after fixation by 4% paraformaldehyde, and the antibodies were incubated with cell suspensions for 20 min in dark place at 4 °C. For nuclear staining, after fixation and permeabilization by buffer set, the antibodies were incubated with cell suspensions for 20 min in dark place at 4 °C.

For staining of peripheral blood samples, after staining, red blood cells were lysed for 4 min at room temperature in red blood cell lysing buffer. Cell suspensions were washed with PBS (4 °C) for two times and filtered using a strainer (70 µm) before flow cytometry analysis. Data were analyzed by FlowJo 10.6.2 software.

The surface staining for sorting was the same as that for flow cytometry analysis, except that all the procedure was operated in a sterile environment.

All the antibodies and related reagents are listed in Additional file 2.

Other methods are detailed in Additional file 2.

### Statistical analysis

Statistical analysis was performed by SPSS 20.0 or GraphPad prism 8.0. Two-tailed *p* value < 0.05 was considered statistically significant. Detailed statistical methods were described in the correspondent figure legends.

## Results

### Upregulation of circTMEM181 is related to anti-PD1 therapy resistance and poor prognosis in HCC patients

**Fig. 1:**
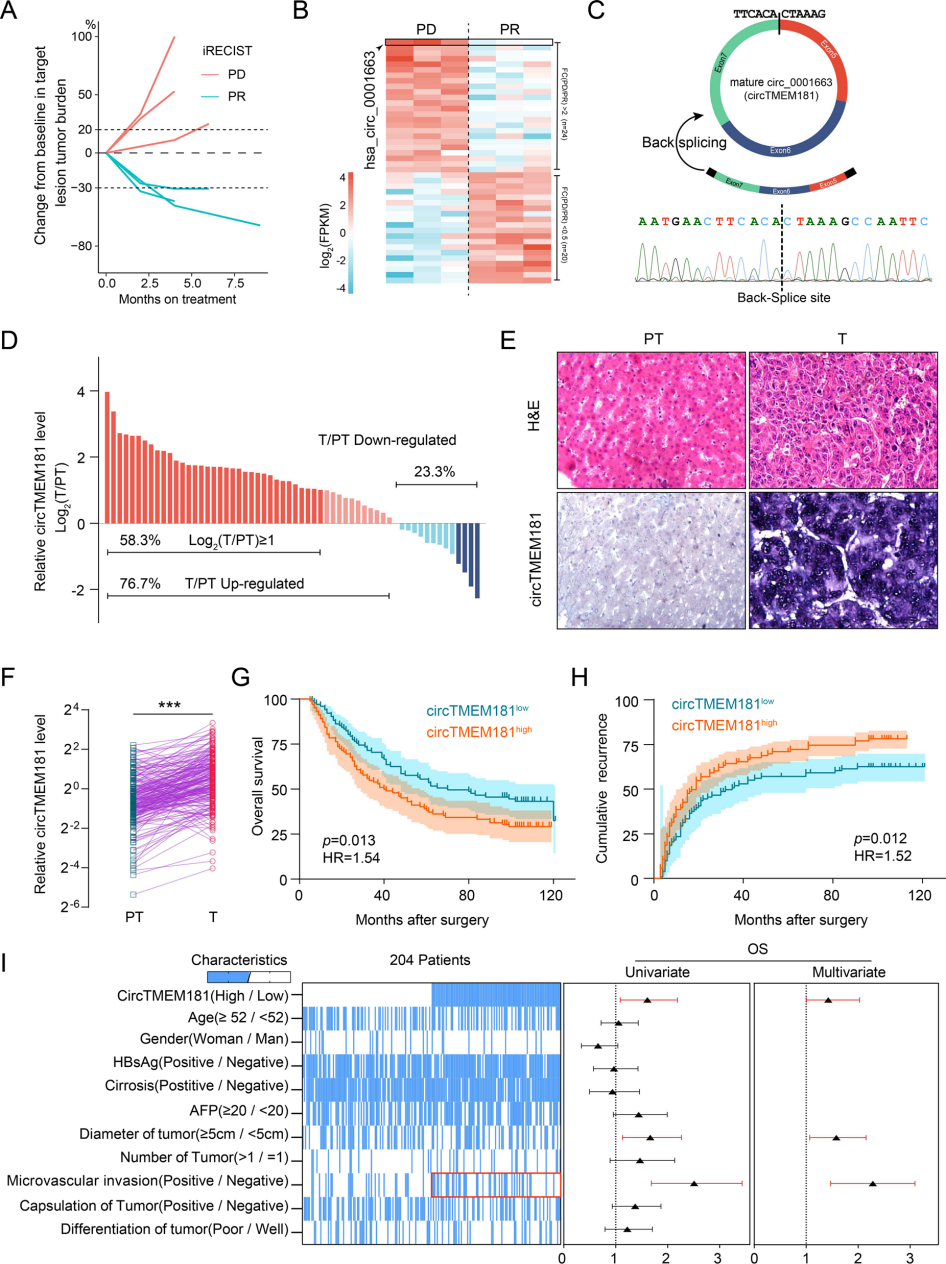
CircTMEM181 is associated with poor prognosis in HCC patients. **a** Changes in tumor size in six patients after anti-PD1 treatment, evaluated by iRECIST. The blue line represents PR (partial response, n = 3), and the orange line represents PD (progressive disease, n = 3). **b** Heatmap of circRNA expression in tumor tissue biopsies from HCC patients showing PR or PD after anti-PD1 treatment. **c** Schematic illustration of circTMEM181 formation (Top). Sanger sequencing shows the back-splice sites (Bottom). **d** A total of 60 paired HCC tumor tissues (T) and para-tumor normal liver tissue (PT) were subjected to qPCR to detect circTMEM181 expression. **e, f** Representative images of H&E staining and in situ hybridization analysis of circTMEM181 expression in HCC tumor tissue or para-tumor normal liver. Statistical results show a higher level of cicTMEM181 in HCC tumor tissue (T) than in the paired para-tumor normal liver (PT) (n = 204; paired t test, ***: *p* < 0.001). **g**, **h** Kaplan–Meier estimate of overall survival or cumulative recurrence in the cohort with different levels of cirTMEM181 (n = 204, logrank test). **i** Heatmap of 204 HCC patients with their clinicopathologic characteristics grouped by circTMEM181 expression (blue indicates the high or positive group of the characteristic; the red frame indicates microvascular invasion and shows significant positive correlation to circTMEM181). Forest plot of univariate or multivariable Cox proportional hazard regression indicates the impact of different characteristics on overall survival (OS)

To investigate the role of circRNAs in HCC patients treated with anti-PD1, 6 patients with advanced HCC treated with nivolumab (human anti-PD1 antibody, Bristol Myers Squibb) were analyzed based on retrospective data. Of these patients, 3 were evaluated as ‘partial response (PR)’ by iRECIST[21] and defined as having anti-PD1-sensitive HCCs, while the others showed ‘progressive disease (PD)’ and were defined as having anti-PD1-resistant HCCs (Fig. 1a). Tumor samples from these 6 patients were collected by puncture biopsy before immunotherapy and were used to perform RNA sequencing. We found that 24 circRNAs were upregulated in anti-PD1-resistant HCCs, and 20 circRNAs were downregulated. Among these differentiated circRNAs, hsa_circ_0001663 (circTMEM181) consistently showed the greatest difference between anti-PD1-resistant HCCs and anti-PD1-sensitive HCCs (Fig. 1b, Additional file 1: Fig. S1a). circTMEM181 splices were generated from exons 5, 6, and 7 of *TMEM181* (Fig. 1c). We used a divergent circTMEM181 primer pair to perform polymerase chain reaction (PCR) and amplified the back-spliced products (168 bp) (Additional file 1: Fig. S1b). Head-to-tail splicing in the PCR products was confirmed by Sanger sequencing (Fig. 1c). Then, 60 pairs of HCC and adjacent tissues were used for real-time quantitative PCR (qPCR), showing that circTMEM181 expression in tumor tissues was significantly higher than that in para-tumor tissues (Fig. 1d). This was confirmed by in situ hybridization in a tissue microarray (TMA) including 204 HCC patients (Fig. 1e and f). These 204 patients were grouped into high and low cricTMEM181 expression according to the median. Survival analysis showed that patients with high cricTMEM181 expression had a shorter OS than those with low expression (median survival: 40 ± 7.4 *vs.* 70 ± 18.8 months). Moreover, patients with high cricTMEM181 expression also tended to have early recurrence after operation (median disease-free survival time: 19 ± 2.8 *vs.* 35 ± 9.7 months) (Fig. 1g and h). Interestingly, correlation analysis showed that patients with high circTMEM181 expression had a significantly high rate of microvascular invasion (*p* = 0.01) (Fig. 1i, red frame). Meanwhile, univariate analysis showed that circTMEM181 (high expression), larger tumors (≥ 5 cm), and microvascular invasion were risk factors for OS of HCC patients (Fig. 1i). In multivariate Cox proportional hazards mode, circTMEM181 (high expression), larger tumors (≥ 5 cm), and microvascular invasion were independent prognostic indicators for OS (Fig. 1i). These results indicate that elevated circTMEM181 plays an important role in HCC progression.

### Overexpression of circTMEM181 reshapes the immune microenvironment in HCC

**Fig. 2:**
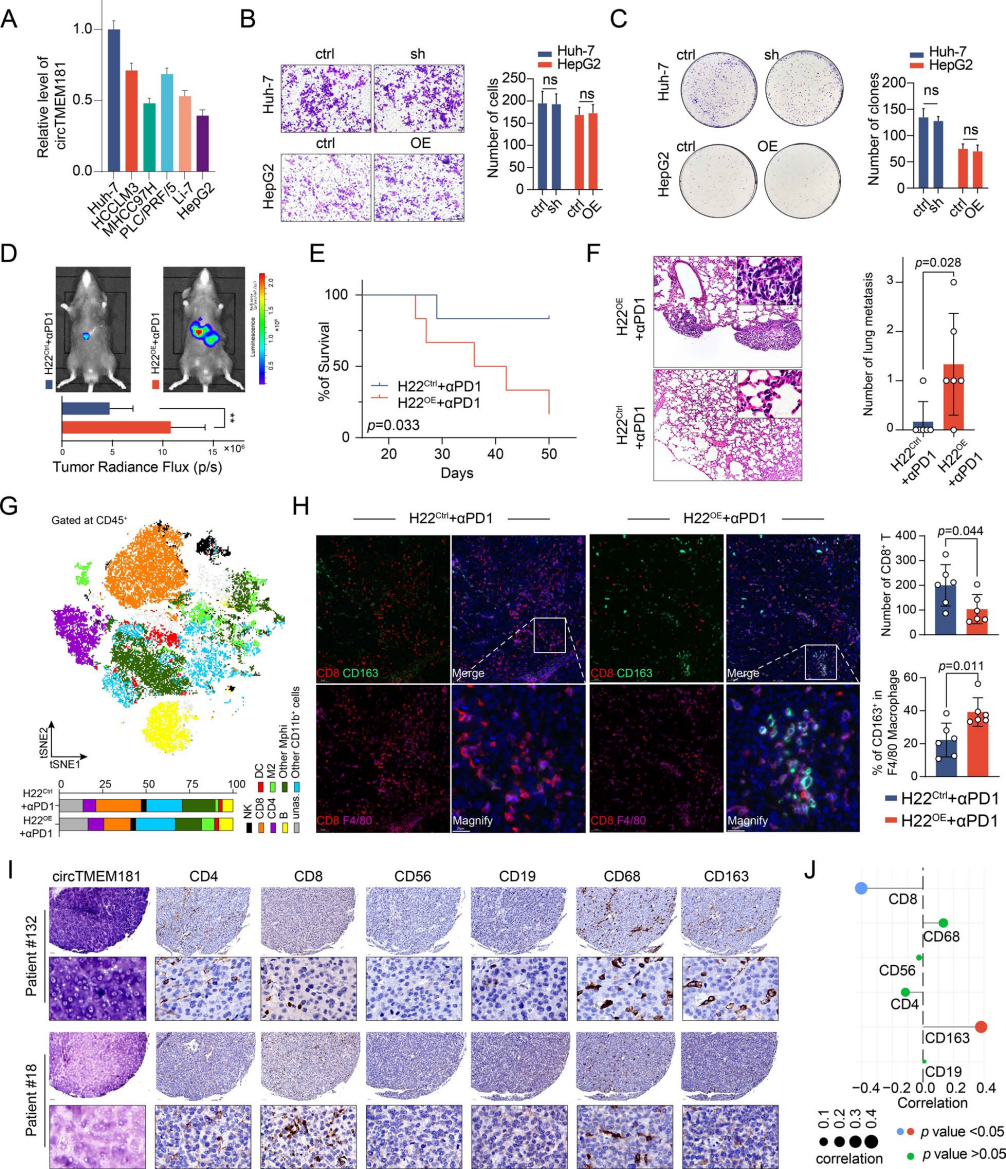
Accumulation of circTMEM181 forms an anti-PD1-resistant immune status in the HCC tumor microenvironment. **a** Levels of circTMEM181 were measured by qPCR in six different HCC cell lines. **b** Representative images of Transwell assays show metastatic ability after circTMEM181 manipulation in the two HCC cell lines (Left). Statistical results of Transwell assays are shown (Right, t test, ns: not significant). **c** Representative images of colony formation assay with crystal violet staining reveal the effects of circTMEM181 on the two HCC cell lines (Left). Statistical results of the colony formation assay are shown (Right, t test, ns: no statistical significance). **d** Representative fluorescence photography of the two groups at 22 days after anti-PD1 therapy in the orthotopic xenograft model of liver cancer (α-PD1: anti-PD1 therapy) (t test, **: *p* < 0.01). **e** Survival curves of six mice per group are shown (logrank test; death or tumor volume > 2.5 cm^3^ were defined as event happened). **f** H22 cell line overexpressing circTMEM181 (H22^OE^) shows more metastatic lung nodules than the control group (H22^ctrl^) in the C57 mouse model (n = 6 mice for each group, t test). **g** tSNE plot and statistical results depict different clusters of CD45^+^ tumor-infiltrating leukocytes from the H22^OE^ and H22^ctrl^ group after anti-PD1 therapy. **h** Representative mIHC images show macrophages and CD8^+^ T cells in tumors of the H22^OE^ and H22^ctrl^ group (Left). Statistical results show less CD8^+^ T cells and more CD163^+^ F4/80^+^ macrophages in the tumor microenvironment in the H22^OE^ group than in the H22^ctrl^ group after anti-PD1 therapy (t test). **i** IHC for CD4 T cells (CD4^+^), CD8 T cells (CD8^+^), NK cells (CD56^+^), B cells (CD19^+^), macrophages (CD68^+^), and M2 macrophages (CD163^+^) from two representative patients with different circTMEM181 expression from our 204 patient cohort. **j** Statistical results show a positive correlation between CD163^+^ macrophages and circTMEM181, but a negative correlation between CD8^+^ T cells and circTMEM181 in our 204 patient cohort (Pearson correlation)

circTMEM181 expression was analyzed in six human HCC cell lines. Among these cells, Huh-7 had the highest circTMEM181 expression, while HepG2 had the lowest level (Fig. 2a). Human HCC cell lines and mouse HCC cell line H22 stably overexpressing or with knocked down circTMEM181 were successfully constructed (Additional file 1: Fig. S1C). Unexpectedly, circTMEM181 expression in HCC cell lines did not influence their metastatic potential or proliferative capacity (Fig. 2b and c). Meanwhile, we also found no statistical difference in the proliferative capacity between H22 cell lines overexpressing circTMEM181 (H22^OE^) and the control (H22^Ctrl^) (Additional file 1: Fig. S2). Next, we investigated the role of circTMEM181 expression in HCC in vivo. H22^OE^ or H22^ctrl^ that were previously labeled with luciferase were used to construct orthotopic liver xenografts in C57 mice. We found that tumors in H22^OE^ + IgG group grow faster than that in H22^ctrl^ + IgG group, indicating that circTMEM181 may promote tumor progression in vivo (Additional file 1: Fig. S3). Interestingly, consistent with our clinical discovery cohort with anti-PD1 therapy (Fig. 1a), H22^ctrl^ was sensitive to anti-PD1 antibody, while H22^OE^ showed a negative response to anti-PD1 antibody therapy (*p* < 0.01) (Fig. 2d). In this orthotopic tumor model with anti-PD1 therapy, OS in the H22^ctrl^ group was also significantly longer than that in the H22^OE^ group (*p* = 0.033) (Fig. 2e). There were significantly more metastatic lung nodules in the H22^OE^ group than in the H22^ctrl^ group (Fig. 2f, right). The sizeable difference between circTMEM181 function in vivo and in vitro indicated that circTMEM181 might mainly affect the immune microenvironment of HCCs rather than tumor cells. Thus, we harvested tumors from the two mouse models that underwent anti-PD1 therapy and analyzed the immune cell composition in CD45^+^ tumor-infiltrating leukocytes (TIL) by flow cytometry. Eight clusters were identified: dendritic cells (DC, CD11b^+^ CD11c^+^), M2 (CD11b^+^ F4/80^+^ CD163^+^), other macrophages (other Mphi, CD11b^+^ F4/80^+^ CD163^−^), CD8^+^ T (CD11b^−^ CD8^+^ CD4^−^), CD4^+^ T (CD11b^−^ CD4^+^ CD8^+^), NK (CD11b^−^ NK1.1^+^), B cells (CD11b^−^ CD19^+^), and CD11b^+^ myeloid cells other than DC or macrophages (Fig. 2g Top). As expected, the H22^OE^ group showed a distinct immune cell profile in the tumor microenvironment compared with the H22^ctrl^ group after anti-PD1 therapy (Fig. 2g Bottom). Particularly, a decreased CD8^+^ T cell population and an increase in F4/80^+^ CD163^+^ M2 macrophages were found in the tumor microenvironment of the H22^OE^ group (Fig. 2g and h). We also investigated CD206 expression (another classical marker for M2 macrophage in mice) by flow cytometry and found an increase in CD206^+^ M2 macrophages in H22^OE^ + αPD1 group (Additional file 1: Fig. S4), consistent with the results that we found in CD163^+^ M2 macrophages. By TMA, we further confirmed that HCC patients with high circTMEM181 expression (circTMEM181^high^) had increased M2 macrophage infiltration (CD163^+^) and decreased CD8^+^ T cell infiltration compared to patients with lower circTMEM181 expression. However, no statistically significant differences in the number of total macrophages (CD68^+^), CD4^+^ T cells, NK cells (CD56^+^), or B cells (CD19^+^) were found (Fig. 2i and j). Our results indicate that overexpression of circTMEM181 promotes HCC development by reshaping the inhibitory immune microenvironment in HCC.

**Fig. 3:**
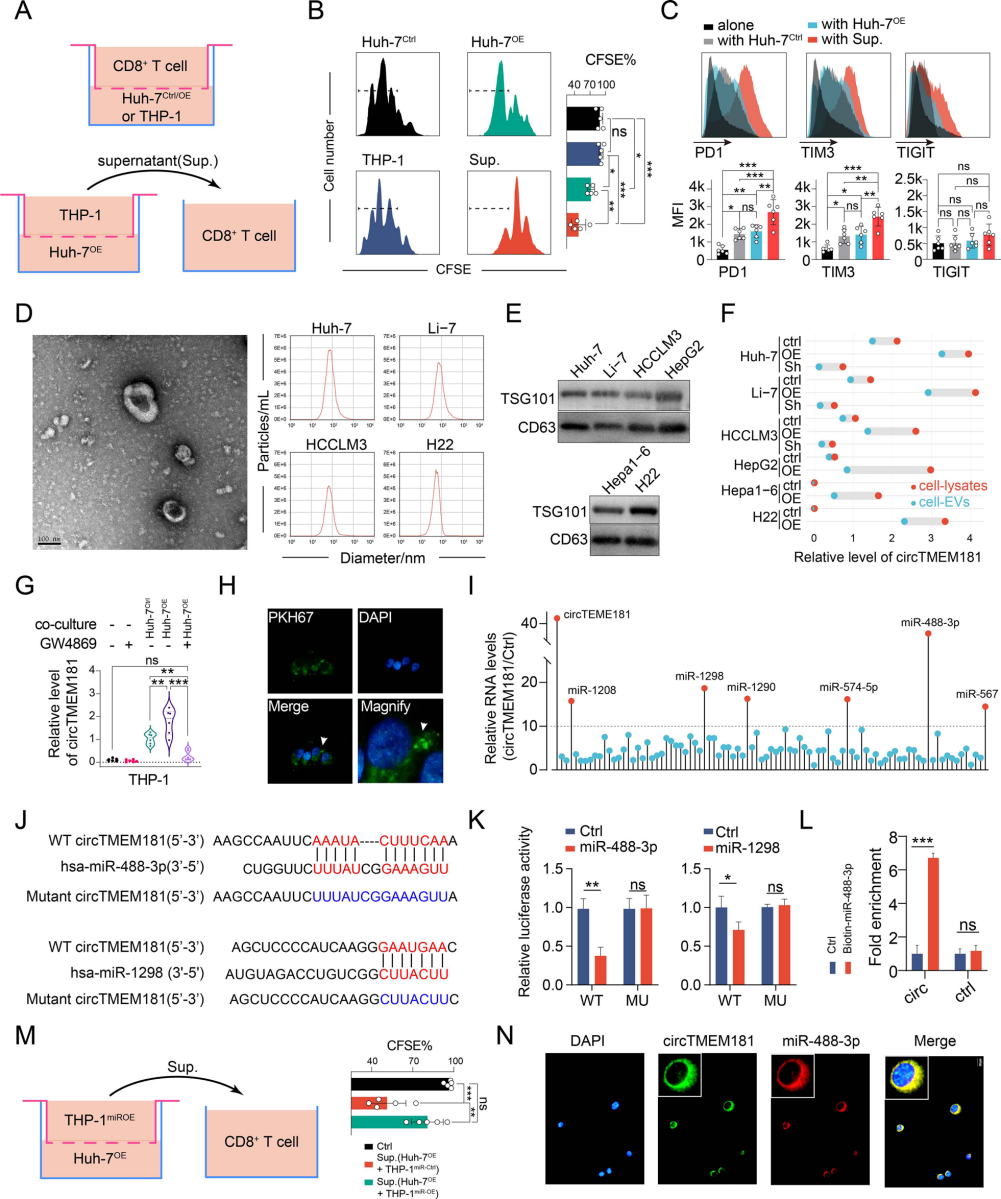
Exosomal circTMEM181 from HCC is internalized by macrophage and sponged miR-488-3p in macrophage. **a** Schematic diagram: CD8^+^ T cells isolated from human PBMCs were co-cultured with Huh-7^circOE^, Huh-7^ctrl^, or THP-1 or the supernatant from THP-1 and Huh-7^OE^ co-culture medium. **b** Flow cytometry analysis was used to evaluate proliferation of CFSE-labeled CD8^+^ T cells in different conditions (Sup.: supernatant of THP-1 and Huh-7^OE^ co-culture medium; one-way ANOVA: ***: *p* < 0.001, **: *p* < 0.01, *: *p* < 0.05, ns: not significant). **c** Flow cytometry analysis of PD1, TIM3, and TIGIT expression on CD8^+^ T cells from different culture conditions. (MFI, mean fluorescent intensity; one-way ANOVA: ***: *p* < 0.001, **: *p* < 0.01, *: *p* < 0.05, ns: not significant). **d** Representative picture of exosomes enriched using ultracentrifugation from medium of Huh-7 by transmission electron microscopy (Left); Nanoparticle tracking analysis was performed to analyze the size distribution of enriched exosomes from different HCC cell lines (Right). **e** Exosomal markers CD63 and TSG101 were detected on enriched exosomes across four human HCC cell lines and two mouse HCC cell lines. **f** CircTMEM181 expression was analyzed in cell lysates or extracellular vesicles (EVs) across different manipulations of various cell lines (^OE^: overexpressing circTMEM181; ^Sh^: circTMEM181 knock down). **g** CircTMEM181 expression was analyzed in THP-1 macrophages co-cultured with or without Huh-7^ctrl^ or Huh-7^OE^ or GW4869 (one-way ANOVA: ***: *p* < 0.001, **: *p* < 0.01, ns: not significant). **h** Immunofluorescence shows exosomes pre-labeled with PKH-67 (green) from Huh-7^OE^ can be internalized by THP-1 (white arrow). **i** RNA immunoprecipitation with circTMEM181-specific probes shows enrichment of RNAs in THP-1 overexpressing circTMEM181 (THP-1^circOE^) compared to the control (THP-1^ctrl^). **j** Schematic diagram: putative binding sites of wild-type circTMEM181, hsa-miR-488-3p, hsa-miR-1298 and mutant circTMEM181. **k** Detection of wild-type circTMEM181-labeled luciferase (WT) or mutant circTMEM181-labeled luciferase (MU) activity in HEK293T cells after miR-488-3p or miR-1298 transfection (t test, *: *p* < 0.05, **: *p* < 0.01, ns: not significant). **l** RNA immunoprecipitation with circTMEM181-specific probes performed in HEK293T cells using biotin-labeled miR-488-3p mimics and a negative control (NC). **m** CD8^+^ T cells isolated from human PBMCs were co-cultured with supernatant of medium from co-culturing Huh-7^OE^ and THP-1 overexpressing miR-488-3p (THP-1^miR−OE^). **n** FISH analysis of circTMEM181 (green) and miR-488-3p (red) demonstrates their colocalization in the THP-1 cytoplasm

**Fig. 4:**
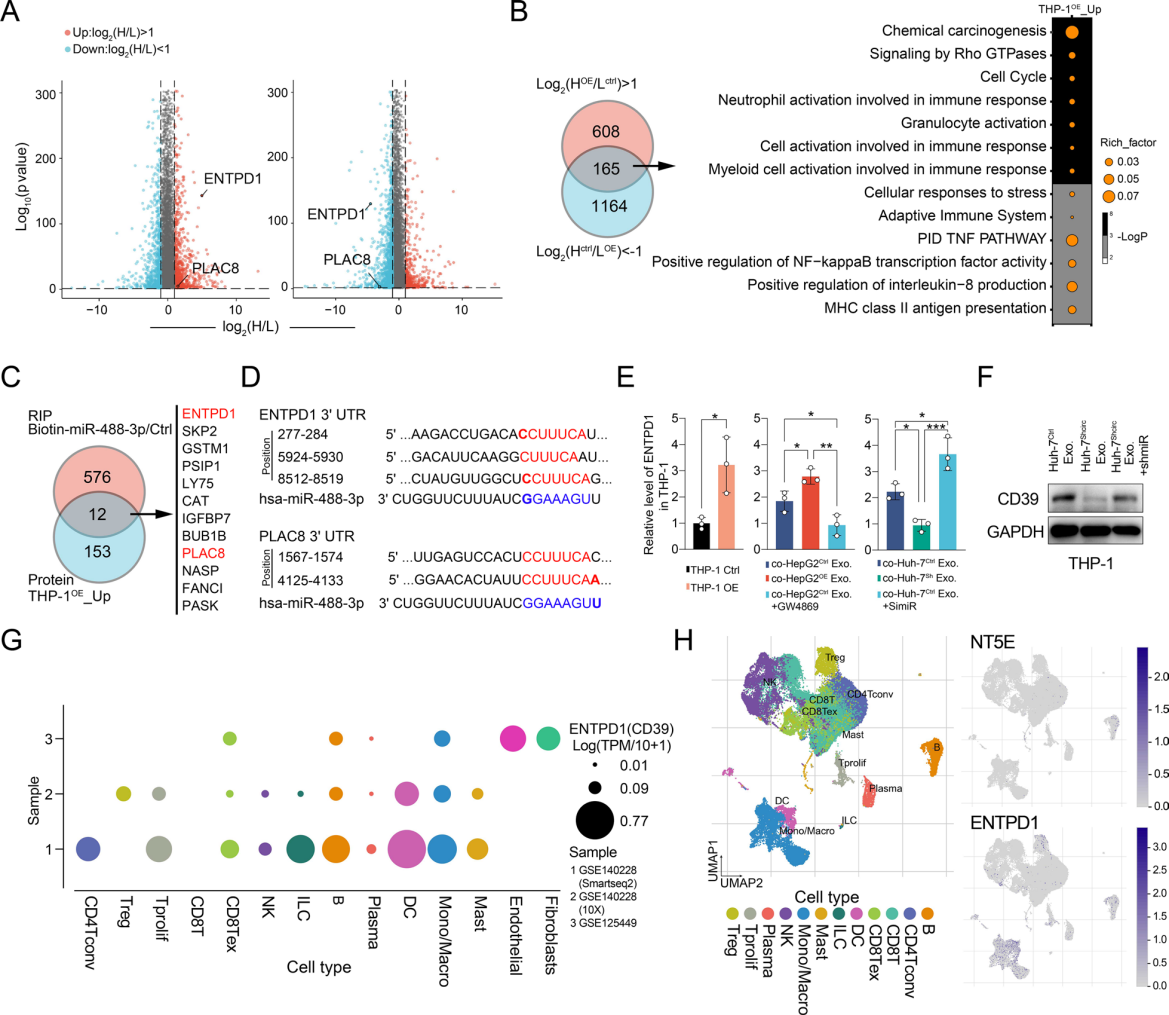
Multi-omics analysis identifies CD39 expression as a target in the circTMEM181-miR-488-3p axis in macrophages. **a** Volcano plot shows varied protein profiles between heavy (H) and light (L) medium. Significantly upregulated proteins in the heavy group are colored in red, while significantly down-regulated proteins in light group are in blue. THP-1 co-cultured with exosomes from HepG2^OE^ (THP-1^co−exoOE^) and paired THP-1 co-cultured with exosomes from HepG2^Ctrl^ (THP-1^co−exoCtrl^) were subjected to SILAC. THP-1^co−exoOE^ were co-cultured with heavy medium (H^OE^), and THP-1^co−exoCtrl^ with light medium (L^Ctrl^) (Left). THP-1^co−exoCtrl^ were co-cultured with heavy medium (H^Ctrl^), and THP-1^co−exoOE^ with light medium (L^OE^) (Right). **b** Venn diagrams show that 165 proteins were upregulated in the intersection of these two forward–reverse experiments (Left). Pathway enrichment of the differentiated proteins according to KEGG and GO analysis (Right). **c** Venn diagrams show 12 mRNAs in the overlap of mRNAs that miRNA 488-3p can target and upregulated proteins in THP-1^circOE^. **d** ENTPD1 and PLAC8 connect to miR-488-3p by TargetScan prediction. **e, f** The level of ENTPD1 was detected in different conditions manipulating circTMEM181, GW4869, or miR-488-3p (t test, ***: *p* < 0.001, **: *p* < 0.01, *: *p* < 0.05). **g** Summary of three scRNAseq samples of human HCC tumor tissues from GEO datasets (GSE140228 (10 ×), GSE140228 (Smartseq2) ,and GSE125449). Monocytes/macrophages in the HCC tumor microenvironment show high expression of CD39 across the three datasets. **h** UMAP analysis showing scRNAseq data of different tumor-infiltrating immune cells in HCC (GSE140228 (10 ×)). Tumor-infiltrating macrophages express a high level of CD39 (ENTPD1) but do not express CD73 (NT5E)

### HCC cell-derived exosomal circTMEM181 targets macrophages to induce the immunosuppressive microenvironment

To further elucidate the mechanism of influence of HCC cell-derived circTMEM181 in tumor-infiltrating immune cells, CD8^+^ T cells isolated from human peripheral blood mononuclear cells (PBMCs) were co-cultured with HCC cells (Fig. 3a). When co-cultured with Huh-7 cells overexpressing circTMEM181 (Huh-7^OE^) for 72 h, CD8^+^ T lymphocytes showed a slight but statistically inhibited proliferation ability compared to the control (Fig. 3b). Given that macrophages were significantly influenced by circTMEM181 expression (Fig. 2g-j), Huh-7 cells were co-cultured with THP-1 macrophages for 72 h, and then the supernatant was collected and incubated with CD8^+^ T cells. Interestingly, CD8^+^ T lymphocytes cultured with the THP-1 and Huh-7^OE^ co-culture supernatant (Sup.) showed obviously inhibited proliferation ability compared to those co-cultured with Huh-7^OE^ or THP-1 (Fig. 3b). Immune checkpoint molecule PD1 and T cell immunoglobulin mucin family member 3 (TIM3) expression was upregulated on CD8^+^ T cells after co-culturing with THP-1 and Huh-7^OE^ supernatant, but we found no different expression of PD1 or TIM3 in CD8^+^ T cells between co-culturing with Huh-7^OE^ and co-culturing with Huh-7^Ctrl^ (Fig. 3c). These results reveal that overexpressing circTMEM181 in HCC cells can affect immune cell status in vitro*.* HCC cells might interfere with the proliferation of CD8^+^ T cell and induce their exhaustion by interacting with macrophages.

Previous studies denoted the importance of exosomes in the interrelationship between tumor and immune cells [22, 23]. Given that the in vitro co-culture system separated tumor cells from PBMCs in independent layers (Fig. 3a), we ruled out the possibility that tumors overexpressing circTMEM181 directly influenced immune cells. Thus, secretory exosomes in media from various HCC cell lines were enriched using ultracentrifugation. The structure and size distribution of enriched exosomes were analyzed by transmission electron microscopy and nanoparticle tracking analysis (Fig. 3d). Exosomal markers CD63 and TSG101 were also detected on enriched exosomes from human and mouse HCC cell lines (Fig. 3e). The circTMEM181 expression level in enriched exosomes was positively related to circTMEM181 expression in corresponding HCC cells (Fig. 3f). Similarly, circTMEM181 expression in the exosome was upregulated when circTMEM181 was overexpressed in HCC cells (Fig. 3f). Further, THP-1 cells expressed slight circTMEM181, which was significantly enhanced when they were co-cultured with Huh-7^ctrl^ or Huh-7^OE^ (Fig. 3g). In addition, circTMEM181 expression in THP-1 cells was largely decreased upon addition of exosome generation inhibitor GW4869 (Fig. 3g). Next, we co-cultured THP-1 cells with exosomes from Huh-7^OE^ pre-labeled with PKH-67. An immunofluorescence assay confirmed that THP-1 cells could internalize exosomes from Huh-7^OE^ (Fig. 3h). This indicates that HCC cells secrete exosomal circTMEM181 and influence macrophage function.

### circTMEM181 sponges miR-488-3p in macrophages

Sponging and decoying miRNAs is a major mechanism by which circRNAs regulate cell functions [14]. Here, we performed RNA immunoprecipitation (RIP) to identify miRNAs that directly interact with circTMEM181. Biotin-circTMEM181-specific probes were transfected into THP-1 cells (THP-1^circOE^), using transfection of biotin alone as a control (THP-1^ctrl^), and RNA sequencing was performed followed by RIP (Fig. 3i). More than tenfold enriched miRNAs were identified, and miR-488-3p and miR-1298 had the top two scores and were selected for further verification (Fig. 3i and j).

Wild-type circTMEM181 (circTMEM181^WT^) or mutant circTMEM181 (circTMEM181^MU^) without miR-488-3p or miR-1298 binding sites were cloned into pLG3 vectors (Fig. 3j). Luciferase reporter activity was then examined with vector-transfected 293 T cells. Our results showed that miR-488-3p and miR-1298 mimic reduced luciferase activity in the circTMEM181^WT^ group but not in the circTMEM181^MU^ group (Fig. 3k). Interestingly, miR-488-3p exerted even less luciferase activity in the circTMEM181^WT^ group than miR-1298. Additionally, biotinylated miR-488-3p could pull down much more circTMEM181 than biotinylated miR-1298 (Fig. 3l), indicating that miR-488-3p has a direct interaction with circTMEM181.

Flow cytometry analysis of CD8^+^ T lymphocytes co-cultured with THP-1 and Huh-7^OE^ showed that the inhibited proliferation ability of CD8^+^ T lymphocytes could be rescued by manipulating miR-488-3p in THP-1 (Fig. 3m). Further, a fluorescent in situ hybridization (FISH) assay showed circTMEM181 colocalized with miR-488-3p in the cytoplasm of THP-1^circOE^ (Fig. 3n).

### circTMEM181 upregulates CD39 expression in macrophages by sponging with miR-488-3p

To detail the function and mechanism of circTMEM181 in macrophages, we performed stable isotope labeling by amino acids in cell culture (SILAC) to compare the protein profiles of THP-1 cells co-cultured with exosomes from HepG2^OE^ (THP-1^co−exoOE^) or HepG2^Ctrl^ (THP-1^co−exoCtrl^) (Fig. 4A). In total, 165 upregulated proteins were found in THP-1^co−exoOE^ (|Log_2_(H/L)|> 1), compared to THP-1^co−exoCtrl^ (Fig. 4b). KEGG and GO analysis showed that the upregulated proteins were majorly involved in immune-associated pathways such as neutrophil activation involved in immune response, myeloid cell activation involved in immune response, and the adaptive immune system (Fig. 4b). Additionally, we used biotinylated miR-488-3p to immunoprecipitate mRNAs from THP-1^co−exoOE^ and identified 588 mRNAs as potential targets of miR-488-3p (Fig. 4c). By overlapping the potential targets with the upregulated proteins, 12 genes were identified as potential molecules downstream of the circTMEM181-miR-488-3p axis (Fig. 4c). Among them, CD39 (ectonucleoside triphosphate diphosphohydrolase 1, ENTPD1) and PLAC8 (placenta specific 8) had more than two positions paired with miR-488-3p (Fig. 4d). ENTPD1, with a high fold change in THP-1^co−exoOE^/THP-1^co−exoCtrl^, was chosen for further study because it is involved in immune-associated function (Fig. 4a).

We found an enhanced level of ENTPD1 mRNA in THP-1 after forced circTMEM181 (THP-1^circOE^) expression (Fig. 4e), verifying the relationship between circTMEM181 and CD39 in macrophages. THP-1 also expressed a high level of CD39 when cultured with exosomes from HepG2^OE^ (co-HepG2^OE^ Exo.), but the upregulation of CD39 could be rescued by administering GW4869 (Fig. 4e). Similarly, the level of CD39 in THP-1 was upregulated after culturing with exosomes from Huh7^Ctrl^ (co-Huh-7^Ctrl^ Exo.) and could be reversed after supplementing with simiR-488-3p (co-Huh-7^Sh^ Exo. + simiR) (Fig. 4e and f).

CD39 is a key enzyme in initiating activation of the ATP–adenosine pathway. By hydrolyzing extracellular ATP (eATP) and ADP to AMP, CD39 can weaken the signal of the immune reaction stimulated by eATP in the tumor microenvironment. Analysis of 50 paired HCC and corresponding normal tissue from TCGA showed that CD39 was overexpressed in HCC tissues (Additional file 1: Fig. S6A). HCC patients with high CD39 expression also showed a poorer OS (Additional file 1: Fig. S6B). We also analyzed scRNA-seq data from three GEO datasets (GSE140228 (10X), GSE140228 (Smartseq2) and GSE125449) and found that tumor-infiltrating macrophages expressed a high degree of CD39 (ENTPD1) among all immune cell types (Fig. 4g and h).

### Increased level of CD39 is implicated in poor prognosis of HCC patients

**Fig. 5:**
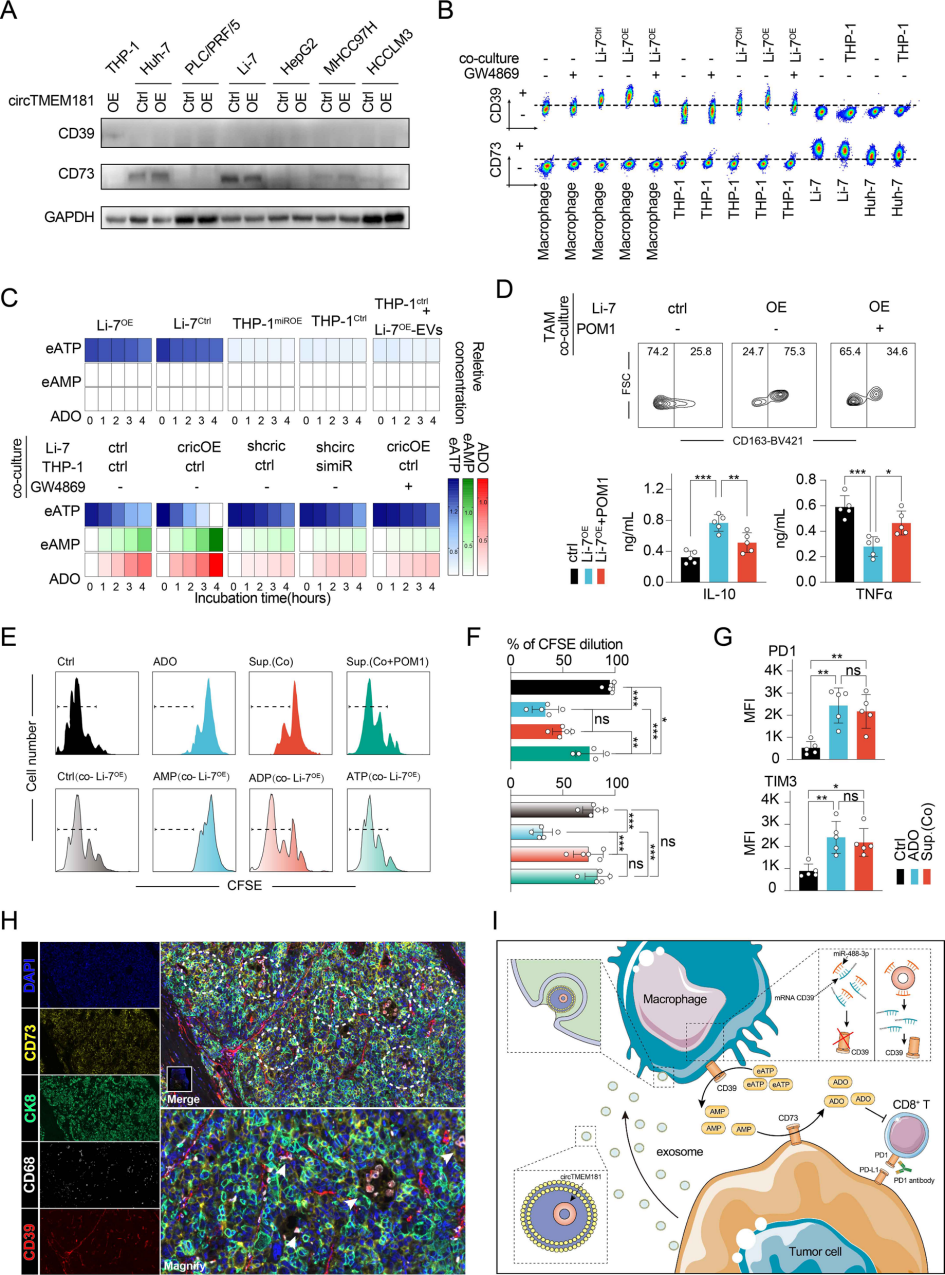
Spatially isolated activation of the ATP–adenosine pathway by macrophages and HCC cell cooperation impairs antitumor immunity. **a** WB analysis shows CD39, CD73, and GAPDH expression across the six HCC cell lines (Huh-7, PLC/PRF/5, Li-7, HepG2, 97H, and HCCLM3) and THP-1^circOE^. **b** Flow cytometry analysis shows CD39 expression on THP-1 or TAM increased after co-culturing with Li-7^OE^, but was rescued after incubating with GW4869. CD73 was detected on Li-7^OE^ co-cultured with/without THP-1, but not detected on TAM or THP-1 with/without co-culturing conditions. **c** Heatmap shows the level of eATP, AMP, and ADO in medium with different conditions. **d** Detection of the function of sorted TAM after co-culturing with Li-7^OE^ or with CD39 inhibitor POM-1. TAM showed an M2 polarization, expressing higher CD163, secreting more IL-10 and less TNFα after co-culturing with Li-7^OE^ (one-way ANOVA: ***: *p* < 0.001, **: *p* < 0.01, *: *p* < 0.05). **e, f** Flow cytometry analysis and statistics show proliferation of CFSE-labeled CD8^+^ T cells in medium with different conditions. Top left to right: CD8^+^ T cells cultured with the supernatant from THP-1 (Ctrl), CD8^+^ T cells cultured with ADO (ADO), CD8^+^ T cells cultured with the supernatant from THP-1 and Li-7^OE^ co-cultured medium (SPNT(Co)), CD8^+^ T cells cultured with the supernatant from THP-1 and Li-7^OE^ co-cultured medium pre-adding POM1 (SPNT(Co + POM1)). Bottom left to right: CD8^+^ T cells cultured with Li-7^OE^ (Ctrl(co-Li-7^OE^)), CD8^+^ T cells cultured with Li-7^OE^ and AMP (AMP(co-Li-7^OE^)), CD8^+^ T cells cultured with Li-7^OE^ and ADP (ADP(co-Li-7^OE^)), CD8^+^ T cells cultured with Li-7^OE^ and ATP (ATP(co-Li-7^OE^)) (one-way ANOVA: ***: *p* < 0.001, **: *p* < 0.01, *: *p* < 0.05, ns: not significant). **g** Flow cytometry analysis of PD1, TIM3 expression on CD8^+^ T cells with/without ADO. (MFI, mean fluorescent intensity; t test: **: *p* < 0.01, *: *p* < 0.05). **h** Multi-label immunofluorescence showing CD39 expression (red) on CD68^+^ macrophages (purple), and CD73 expression (yellow) on CK8^+^ HCC tumor cells (Green) in the HCC tumor microenvironment (white arrow indicates spatial isolated CD73^+^ CK8^+^ HCC cells and CD39^+^ CD68^+^ macrophages; dotted line circle indicates the niches where CD39^+^ CD68^+^ macrophages are surrounded by CD73^+^ CK8^+^ HCC tumor cells). **i** Graphical abstract showing the activation of the spatial isolated ATP–adenosine pathway by tumor–macrophage communication in the HCC tumor microenvironment

We next investigated the function of CD39 in HCC. Recent studies have observed a high level of CD39 in tumor-infiltrating immune cells from many tumors [12, 24, 25]. Some tumors including kidney, lung, testicular, and thyroid tumor cells, but not HCC, express a high level of CD39 [26]. Here, we found that THP-1 macrophages, but not HCC cells, expressed a high level of CD39 (Fig. 5 A, Additional file 1: Fig. S6C and D). Immunohistochemistry (IHC) analysis of TMAs showed CD39-positive expression on the cell membrane and cytoplasm of immune cells, vascular endothelial cells, and fibroblasts, but only some tumor cells in tumor tissues (Additional file 1: Fig. S6E). Consistent with the results from TCGA, our data also showed that higher tumor CD39 in HCC patients was associated with poorer prognosis in terms of shorter OS and earlier recurrence (Additional file 1: Fig. S6F and G).

**Fig. 6:**
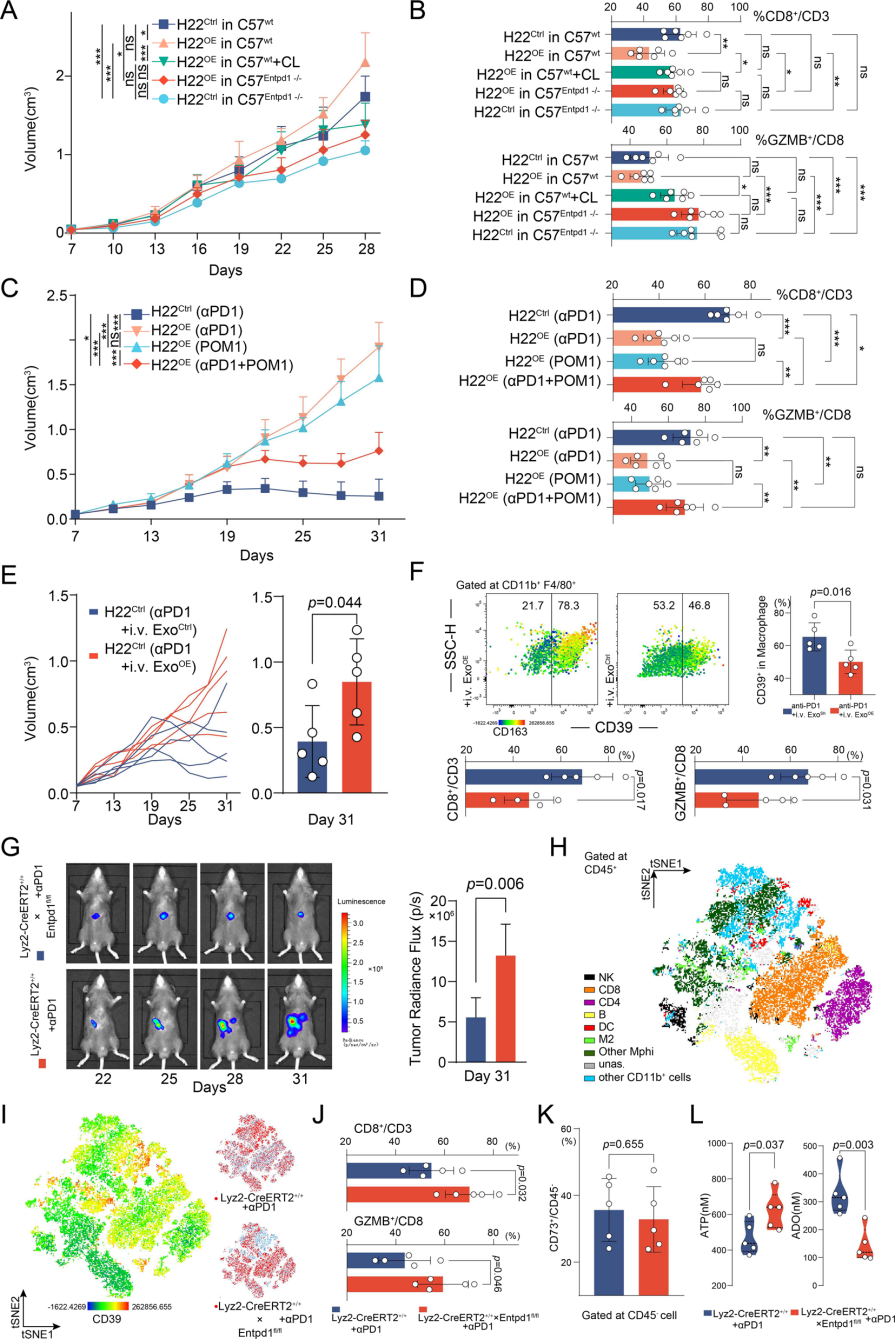
Targeting CD39 on macrophages is a potential therapeutic strategy to overcome immunotherapy resistance in HCC. **a** Tumor size was measured over time after H22^Ctrl^ (H22 overexpressing Control) or H22^OE^ (H22 overexpressing circTMEM181) were used to construct tumor xenografts in the C57^wt^ (C57BL/6 wild-type) or C57^Entpd1−/−^ (C57BL/6 Entpd1-knocked-out) mouse models (n = 6 for each group; one-way ANOVA: ***: *p* < 0.001, **: *p* < 0.01, *: *p* < 0.05, ns: not significant). **b** Flow cytometry analysis shows the proportion of CD8^+^ T cells and GZMB^+^ CD8^+^ T cells in the tumor microenvironment of HCC mouse models (one-way ANOVA: ***: *p* < 0.001, **: *p* < 0.01, *: *p* < 0.05, ns: not significant). **c** Tumor size was measured over time following PD1 antibody (αPD1), POM1 or combination therapy (αPD1 + POM1) in C57 mouse models (n = 6 for each group; one-way ANOVA: ***: *p* < 0.001, ns: not significant). **d** Flow cytometry analysis shows proportion of CD8^+^ T cells and GZMB^+^ CD8^+^ T cells in the tumor microenvironment of HCC mouse models (one-way ANOVA: ***: *p* < 0.001, **: *p* < 0.01, *: *p* < 0.05, ns: not significant). **e** Tumor size was measured over time following treatment with PD1 antibody + infusion of exosome enriched from H22^OE^ medium (αPD1 + i.v. Exo^OE^) or H22 Ctrl medium (αPD1 + i.v. Exo^Ctrl^) in C57^wt^ mouse models (n = 5 for each group; t test). **f** Flow cytometry analysis of the tumor microenvironment indicates a significant increase in CD39 expression on TAM (CD11b^+^ F4/80^+^) after infusion of exosomes enriched from H22^OE^ medium and the CD39^high^ TAM tended to be co-expressed with CD163^high^ (Top). Statistical results show the proportion of CD8^+^ T cells and GZMB^+^ CD8^+^ T cells in the tumor microenvironment of HCC mouse models (t test). **g** Representative fluorescence photography of the two indicated groups at different time points after anti-PD1 therapy in the orthotopic xenograft model of liver cancer (t test, **: *p* < 0.01). **h** tSNE plot of different major clusters in CD45^+^ tumor-infiltrating immune cells from the two indicated groups. **i** tSNE plot of CD39 fluorescence intensity in different clusters between the two indicated groups. **j** Flow cytometry analysis shows proportion of CD8^+^ T cells and GZMB^+^ CD8^+^ T cells in the tumor microenvironment (t test). **k** Flow cytometry analysis shows CD73 expression on CD45^−^ H22 tumor cell in the HCC mouse model (t test). **l** Measurement of serum ATP and ADO concentrations 20 days after anti-PD1 therapy in the orthotopic xenograft model of liver cancer (t test)

### Macrophages CD39 and HCC cells CD73 synergistically activate ATP–adenosine pathway to impair antitumor immunity

Extracellular ATP (eATP) is degraded into adenosine (ADO) through an ATP–adenosine pathway. Within the tumor microenvironment, accumulated eATP could promote immune activity. However, upregulated ADO has been found to be related to the tumor immunosuppressive microenvironment, and CD39/CD73 are the two key enzymes mediating the ATP–ADO process. CD39 can degrade eATP into ADP and AMP, and then CD73 degrades AMP into ADO. Interestingly, most HCC cell lines expressed high CD73, and Li-7 expressed the highest level of CD73 among them (Fig. 5a). Notably, CD73 expression on HCC cell lines was in a circTMEM181-independent manner (Fig. 5a). TMA analysis also showed that CD73 expression was ubiquitous in HCC cells (Additional file 1: Fig. S7A). Specially, CD73 was not detected on THP-1^circOE^ cells (Fig. 5a). To address the relationship among CD39 expression in macrophages, CD73 expression in HCC cells, and circTMEM181 expression, we co-cultured THP-1 and TAM (tumor-associated macrophages) isolated from HCC tissues with Li-7 overexpressing circTMEM181 (Li-7^OE^). Flow cytometry analysis showed that CD39 expression in THP-1 or TAM was enhanced after co-culturing with Li-7^OE^, but could be blocked by GW4869 (Fig. 5b). Meanwhile, CD73 was detected on Li-7^OE^, but not on TAM or THP-1, even upon culturing with HCC overexpressing circTMEM181 (Fig. 5B). Public scRNA-seq data supported our results that macrophages in HCC express a high level of CD39 but a low level of CD73 (Fig. 4h). To eliminate the potential effect of circTMEM181 on soluble CD73 (sCD73), sCD73 was also detected in different conditions, but no significant differences of sCD73 were observed (Additional file 1: Fig. S7B and C).

**Fig. 7:**
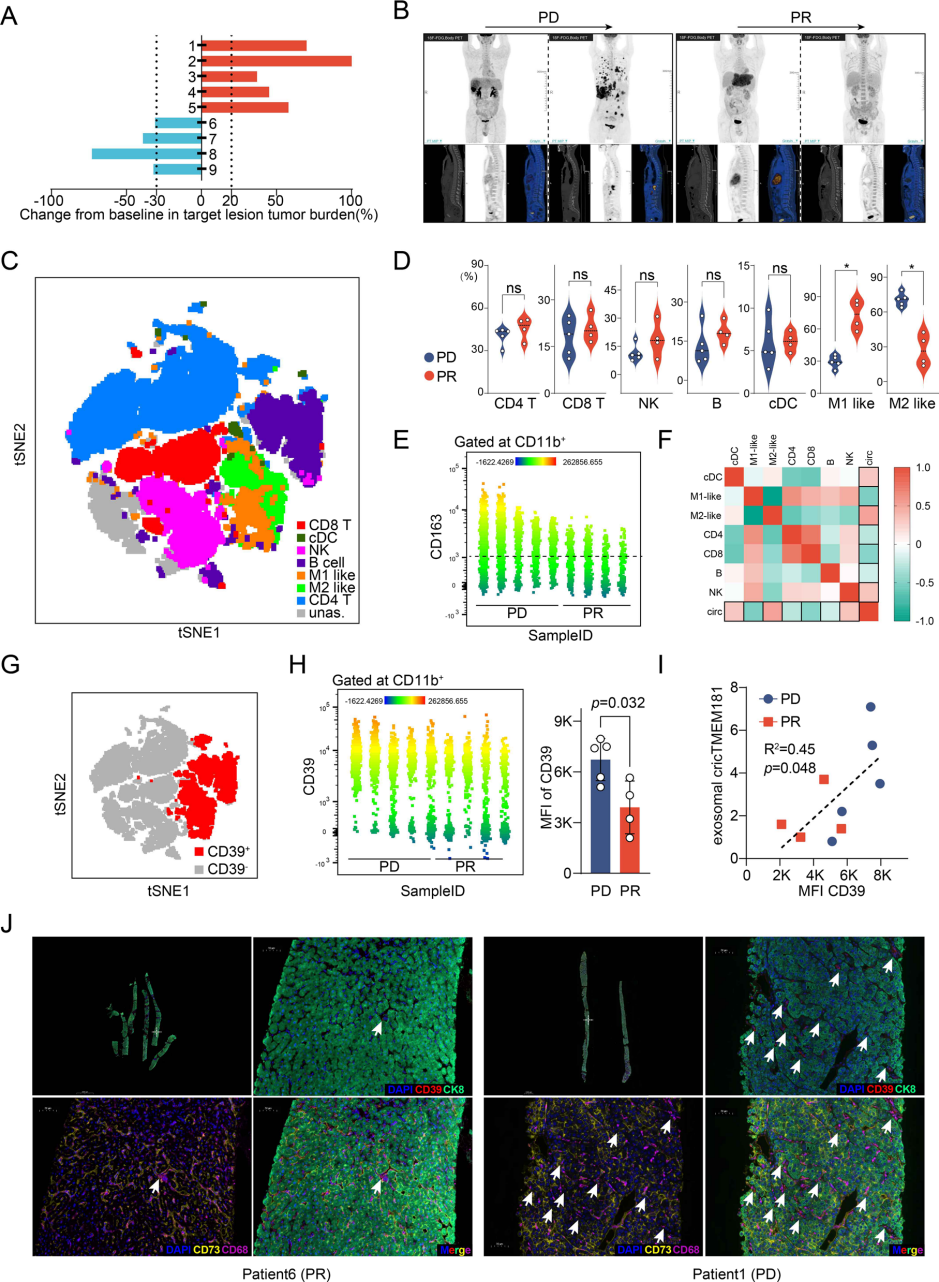
HCC patients whose spatial isolated ATP–adenosine pathway was activated by tumor–macrophage communication show poor response to PD1 antibody therapy. **a** Bar plot indicating the change in tumor size in the nine patients after treatment with PD1 antibody nivolumab. Evaluation by iRECIST, the blue bar represents PR (partial response, n = 4) and the red line represents PD (progressive disease, n = 5). **b** Representative PET-CT results show tumor size of two patients before and after PD1 antibody therapy (left patient: PD; right patient: PR). **c** tSNE plot of different major immune cell clusters in PBMCs from the nine patients (CD4 T: CD11b^−^ CD4^+^ CD8^−^ cells; CD8 T: CD11b^−^ CD4^−^ CD8^+^ cells; NK: CD11b^−^ CD56^+^ cells; B cell: CD11b^−^ CD19^+^ cells; cDC: CD11b^−^ CD141^+^ cells; M1 like: CD11b^+^ CD163^−^ cells; M2 like: CD11b^+^ CD163^+^ cells; unas.: unassigned cells). **d** Violin plot of different immune cell clusters in PBMCs from the nine PD, PR HCC patients after anti-PD1 therapy (CD4 T%: rate of CD4^+^ CD8^−^ cells in gated CD11b^−^ cells; CD8 T%: rate of CD4^−^ CD8^+^ cells in CD11b^−^ gated cells; NK%: rate of CD56^+^ cells in gated CD11b^−^ cells; B %: rate of CD19^+^ cells in gated CD11b^−^ cells; cDC %: rate of CD141^+^ cells in gated CD11b^−^ cells; M1 like%: rate of CD163^−^ cells in gated CD11b^+^ cells; M2 like%: rate of CD163^+^ cells in gated CD11b^+^ cells; Mann–Whitney test: *: *p* < 0.05, ns: not significant). **e** Fluorescence intensities of CD163 (PE-cy7) in CD11b^+^ cells from the nine patients are displayed as heatmap statistics by flow cytometry analysis. **f** Heatmap shows the correlation trends between level of exosomal circTMEM181 in blood serum and different immune cell clusters (Spearman correlation coefficient is indicated by heatmap). **g** tSNE plot of CD39^+^ cell clusters (red) in PBMCs from the nine patients. **h** Fluorescence intensities of CD39 (PE) in CD11b^+^ cells from the nine patients are displayed as heatmap statistics of flow cytometry analysis (Left). The statistical results show the mean fluorescence intensity (MFI) difference between the PD and PR groups among the nine patients (Right; Mann–Whitney test: *p* = 0.032). **i** Correlation between level of exosomal circTMEM181 in blood serum and MFI of CD39 (PE) in CD11b^+^ cells from PR or PD patients (simple linear regression: R^2^ = 0.45, *p* = 0.048). **j** Representative multi-label-immunofluorescence image showing CD39 expression on macrophages (CD68^+^), and CD73 expression on tumor cells (CK8^+^) in the tumor microenvironment of PD or PR HCC patients after PD1 antibody therapy

To confirm how this CD39 (mostly expressed on macrophages)–CD73 (mostly expressed on HCC cells) axis affects the ATP–adenosine pathway in the tumor environment, we then monitored the level of eATP, AMP, and ADO in medium with different conditions. Only ample eATP, but not AMP or ADO, was detected in the medium of Li-7^OE^ or THP-1 cultured alone (Fig. 5c). Adding exosomes from Li-7^OE^ also could not elevate the ADO level in the medium when culturing THP-1 alone (Fig. 5c). However, when co-culturing THP-1 and Li-7^OE^, the level of eATP decreased and AMP and ADO were increased in the medium (Fig. 5c). The increase in ADO and AMP in the co-culturing conditions could be largely inhibited by adding GW4869 or overexpressing miR-488-3p in THP-1 (THP-1^miROE^) (Fig. 5c). We also investigated the function of TAM after co-culturing with Li-7^OE^ or CD39 inhibitor polyoxometalate 1 (POM1) (Fig. 5d). Interestingly, TAM showed a M2-like polarization characterized by high CD163 expression, more secretion of IL-10, and less secretion of TNF-α after co-culturing with Li-7^OE^ (Fig. 5d). These findings manifest that the interaction between HCC cells and THP-1 macrophages is involved in activation of the ATP–adenosine pathway and induces macrophage M2-like polarization.

We further tested the role of ADO in CD8^+^ T cell exhaustion. Co-cultured with ADO, CD8^+^ T cells showed high PD1 and TIM-3 expression and their activity was inhibited (Fig. 5E, F, and G). Similar results were observed in CD8^+^ T cells cultured with the supernatant from co-culture of macrophages and Li-7^OE^ (Sup.(Co)), but this inhibition could be rescued by culturing with the supernatant from co-culture of macrophages and Li-7^OE^ pre-adding POM1 (Sup.(Co + POM1)) (Fig. 5e, f, and g). However, when co-cultured with Li-7, only adding AMP, but not ADP or ATP, CD8^+^ T cells showed inhibited proliferation, indicating that HCC cells only degraded AMP into ADO by CD73 in the absence of CD39 expression (Fig. 5e and f). We then performed multiplex immunohistochemistry (mIHC) in HCC tissue to confirm CD39 and CD73 expression in the HCC microenvironment (Fig. 5h and Additional file 1: Fig. S8). CD39 was mostly expressed on endothelial cells, macrophages (CD68^+^), and some other immune cells (CD68^−^), but was not expressed on tumor cells (CK8^+^). As expected, CD73 was mostly expressed on CK8^+^ tumor cells, but barely expressed on macrophages (CD68^+^) (Fig. 5h and Additional file 1: Fig. S7D). Interestingly, CD39^+^ CD68^+^ macrophages were surrounded by CD73^+^ CK8^+^ HCC cells, indicating activation of the ATP–adenosine pathway by collaboration of tumors and macrophages in the HCC microenvironment (Fig. 5h).

### Depletion of CD39 or macrophages inhibits HCC progression and CD8^+^ T cell exhaustion in mice

The above data showed that HCC-cell-derived exosomal circTMEM181 enhanced CD39 expression in macrophages. In collaboration with CD73 on HCC, elevated CD39 degraded eATP into ADO, impairing the function of CD8^+^ T cells (Fig. 5i). To confirm these findings in vivo, we constructed subcutaneous tumor xenograft models in C57^wt^ mice and C57 mice with deleted CD39 (C57^Entpd1−/−^). Moreover, Clodronate liposomes (CL) were also used to delete macrophages in the tumor microenvironment.

We found that the tumor volume of H22^OE^ was larger than that of H22^Ctrl^ in C57^wt^ mice (Fig. 6a). Meanwhile, no statistical difference in tumor volume was found between the CL group (H22^OE^ in C57 treated with CL) and the control group (H22^Ctrl^ in C57) (Fig. 6a). In C57^Entpd1−/−^, both H22^OE^ and H22^Ctrl^ grew slower than that in the C57^wt^ model, but the tumor volume from H22^OE^ or H22^Ctrl^ showed no statistical difference (Fig. 6a). Tumors harvested from H22^OE^ in C57 mice showed the lowest infiltration of CD8^+^ T cells and the lowest granzyme B expression on CD8^+^ T cells among all the groups (Fig. 6b). Although deletion of CD39 in C57 mice did not increase the infiltration of CD8^+^ T cells compared with the control group (H22^Ctrl^ in C57), we found a significant increase in the granzyme B level on CD8^+^ T cells in the Entpd1^−/−^ groups (Fig. 6b).

### Targeting macrophage CD39 is a potential therapeutic strategy to reverse anti-PD1 resistance in HCC

Presently, several clinical trials targeting CD39 are ongoing (ClinicalTrials.gov identifiers: NCT03884556, NCT04261075, NCT04336098). Given the role of the cricTMEM181–macrophage ^CD39^–HCC ^CD73^ axis in tumor growth and CD8^+^ T cells, we then explored whether interfering with this axis, especially CD39, can enhance the efficiency of PD1 immunotherapy.

Subcutaneous tumor xenografts in C57 mice were constructed by injecting H22^OE^ or H22^Ctrl^ cells. Our findings showed a significantly larger tumor volume in the H22^OE^ group than in H22^Ctrl^ after anti-PD1 antibody therapy (Fig. 6c). In addition, H22^OE^ showed limited response to POM1 alone, while tumor growth was significantly inhibited when H22^OE^ were treated with a combination of anti-PD1 antibody and POM1 (Fig. 6c). Harvested tumors showed significant increases in CD8^+^ T cell infiltration and granzyme B expression on CD8^+^ T cells in the combined anti-PD1 antibody and POM1 group compared to the other two H22^OE^ groups treated with anti-PD1 antibody alone or with POM1 alone (Fig. 6d).

However, tumor size largely increased in the H22^Ctrl^ group after intravenous injection of exosomes enriched from H22^OE^ medium (Fig. 6e). A significant increase in CD39 expression on TAM (CD11b^+^ F4/80^+^) after infusion of exosomes enriched from H22^OE^ medium was found in harvested tumor. These CD39^high^ TAM tended to co-express CD163^high^ (Fig. 6f). In addition, significant decreases in CD8^+^ T cell infiltration and granzyme B expression on CD8^+^ T cells were found after infusion of exosomes enriched from H22^OE^ medium (Fig. 6f).

To further confirm whether specific depletion of CD39 on macrophages can enhance the effect of anti-PD1 antibody, C57-Lyz2^CreERT2^ × C57*-*Entpd1^fl/fl^ mice were constructed (Additional file 1: Fig. S9A). Peripheral blood was harvested to verify selective deletion of CD39 on the macrophages after tamoxifen use (Additional file 1: Fig. S9B). H22^OE^-labeled luciferase was then used to construct orthotopic liver xenografts in this model, and the mice were treated with anti-PD1 antibody. Interestingly, H22^OE^ showed poor response to anti-PD1 antibody, but selective deletion of CD39 on macrophages induced significant tumor shrinkage in response to anti-PD1 therapy (Fig. 6g). Flow cytometry analysis of CD45^+^ TIL showed that CD39^high^ was mostly expressed on CD11b^+^ myeloid cells and partially on CD8^+^ and CD4^+^ cells (Fig. 6h and i). Selective deletion of CD39 on macrophages effectively reduced CD39 expression on CD11b^+^ cells (including macrophages) in the tumor microenvironment (Fig. 6i). More CD8^+^ T cell infiltration and granzyme B expression on CD8^+^ T cells were found after selective deletion of CD39 in macrophages (Fig. 6j). Meanwhile, no significant change in CD73 expression was observed in H22 tumor cells after selective deletion of macrophage CD39 (Fig. 6k). Notably, we also found a significantly higher concentration of ADO and lower ATP in mouse serum upon selective deletion of CD39 on the macrophages (Fig. 6l).

### Activation of the ATP–adenosine pathway by interaction of tumor cells and macrophages results in poor response to anti-PD1 therapy in HCC patients

Nine advanced HCC patients evaluated as PD or PR (iRECIST) after receiving the PD1 inhibitor nivolumab were analyzed. Five of the nine patients showed PD after PD1 immunotherapy, and four showed PR (Fig. 7a and b). The circTMEM181 level of the 9 tumor samples collected by puncture biopsy before immunotherapy together with the 6 tumor samples in our previous discovery cohort (Fig. 1a) was analyzed by qPCR. Our data confirmed a significant higher circTMEM181 expression in anti-PD1-resistant HCCs from the 15-patient cohort (Additional file 1: Fig. S10). Blood serum and PBMCs from the nine patients were also collected for further analysis of their immune status and blood exosomes. Seven major clusters were identified in the PBMCs: CD8 T (CD11b^−^, CD4^−^, CD8^+^), CD4 T (CD11b^−^, CD4^+^, CD8^−^), B cells (CD11b^−^, CD19^+^), NK (CD11b^−^, CD56^+^), cDC (CD11b^+^, CD141^+^), M1-like monocytes/macrocytes (CD11b^+^, CD163^−^), and M2-like monocytes/macrocytes (CD11b^+^, CD163^+^) (Fig. 7c). Although most clusters of immune cells remained relatively stable, patients evaluated as PD tended to have more CD11b^+^CD163^+^ M2-like monocytes/macrocytes and less CD11b^+^CD163^−^ M1-like monocytes/macrocytes in their PBMCs (Fig. 7d and e). We then explored the correlation between different immune cell clusters and the level of exosomal circTMEM181 in blood serum. Exosomal circTMEM181 showed a positive correlation tendency with cDCs, M2-like monocytes/macrocytes, and NK cells, but a negative correlation tendency with M1-like monocytes/macrocytes, CD8 T, CD4 T and B cells (Fig. 7f). We also found CD39 expression mostly on CD19^+^ B cells, CD11b^+^ myeloid cells, and some CD4^+^ T cells in the nine patients, and patients with PD expressed a higher level of CD39 on CD11b^+^ myeloid cells than patients with PR (Fig. 7g and h). Importantly, exosomal circTMEM181 in blood serum showed a positive correlation with CD39^+^ cells in PBMCs (Fig. 7i). Diagnostic liver biopsies of four patients before anti-PD1 therapy were analyzed by IHC. Our mIHC showed that CD39 was located on macrophages (CD68^+^), while CD73 was mostly located on HCC cells (CK8^+^) (Fig. 7j). More CD39^+^ macrophages (white arrow) were found in the tumor tissues from patients with PD (Fig. 7j), indicating an activated ATP–adenosine pathway in the tumor microenvironment of PD patients.

## Discussion

Complex compensatory networks resulting from communication between tumor-infiltrating immune cells and tumor cells narrow the effect of immunotherapy, leading to drug resistance and treatment failure [27, 28]. Our study unveiled a new interactive net between HCC cells and tumor-infiltrating macrophages: HCC cells secrete exosomal circTMEM181 to influence macrophages, induce enhanced expression of CD39 on macrophages, and reshape the HCC immune environment. The collaboration of CD39 (in macrophages but not HCC cells) with CD73 (mostly in HCC cells but not macrophages) fulfills the sequential activation of the ATP–adenosine pathway, impairs CD8^+^ T cell function, and finally builds a PD1 antibody-resistant tumor environment. Currently, accumulating evidence uncovers the critical role of circRNA in carcinogenesis and cancer development. However, the function of circTMEM181 in cancer has not been reported. circTMEM181 was generated from exons 5, 6, and 7 of TMEM181, a gene encoding a putative G protein-coupled receptor expressed on the cell surface [29, 30]. In this study, we firstly reveal circTMEM181’s participation in tumor cell-macrophage interaction, and this cross talk finally promotes HCC progression and limits anti-PD1 therapy response. Moreover, our research provides a reasonable rationale for blockading the interaction between macrophages and tumor cells to reverse anti-PD1-resistant HCC.

The efficiency of immunotherapy varies widely in different tumors. For example, patients with melanoma or lung cancer respond better to PD1 antibody than patients with HCC [4–6]. This phenomenon is mainly due to differences in the tumor microenvironment [31]. As the biggest solid tissue, the liver is characterized by a rich blood supply and many rested macrophages [32]. Thus, HCC develops in a complex environment different from that of other tumors. Our study investigated tumor and immune cells in the context of HCC and showed that circTMEM181 is highly expressed in HCC patients with poor response to PD1 antibody. However, circTMEM181 cannot directly promote the malignant phenotype of HCC cells in vitro. Then, we demonstrated that HCC cells secrete circTMEM181 through exosomes. This exosomal circTMEM181 elevates CD39 expression on macrophages by sponging with miR-488-3p in macrophages, leading to changes in macrophage function and in the immune microenvironment (including polarization of M2 and CD8^+^ T cell anergy). A recent study also analyzed microenvironment and immunotherapy response of HCC, demonstrating three different immune clusters. Our findings showed an anti-PD1-resistant status like the Cluster2 in the study, characterized by high infiltration of TAM and low infiltration of cytotoxic lymphocytes [33].

The effects of the ATP–adenosine pathway on tumors or immunity have been studied in detail [12]. Due to hypoxia and tumor cell necrosis in the tumor environment, a large amount of eATP is released and immune cells become activated, suppressing tumor progression. However, adenosine inhibits immune cell activity. In the ATP–adenosine pathway, CD39 metabolizes eATP into ADP and AMP, but cannot further catabolize AMP. On the contrary, CD73 can only further decompose AMP into adenosine, but cannot directly metabolize eATP [12]. Therefore, cooperation between CD39 and CD73 is essential to metabolize eATP into adenosine [34].

Our study found that CD73, but not CD39, is expressed ubiquitously on HCC cells. Therefore, CD39, but not CD73, triggers eATP–adenosine activation in HCC. Our study illustrated a mechanism in which HCC cells subjectively elevate CD39 expression on macrophages by exosomal circRNA. In this setting, more eATP can be hydrolyzed into AMP through elevated CD39 on macrophages. AMP is further hydrolyzed into adenosine by CD73 on the surface of HCC cells. The sequential cooperation of CD73^+^ HCC cells and CD39^+^ macrophages results in adenosine accumulation and suppression of CD8^+^ T cells and creates an immunosuppressive tumor microenvironment resistant to PD1 antibody. Macrophage-specific knockout of CD39 in our mouse model significantly reversed resistance to PD1 therapy, suggesting that targeting CD39 on macrophages may remodel the immune microenvironment and allow HCC patients to re-gain benefit from anti-PD1 therapy.

CD39 is expressed on many immune cells, including CD8^+^ T cells, macrophages, Treg cells, and endothelial cells [12]. Additionally, CD39 is expressed on some tumor cells, including lung cancer cells, melanoma cells, and thyroid tumor cells, but not on HCC cells [26]. Our large multiplex immunohistochemistry cohort confirmed that CD39 is barely expressed on HCC cells but is expressed on CD68^+^ macrophages in HCC tissues. On the contrary, CD73 is hardly expressed on CD68^+^ macrophages, but widely expressed on HCC cells. This ‘spatially isolated’ CD39–CD73 expression is relatively special in the HCC environment. In fact, many studies confirmed that expression of CD39 and CD73 can be regulated by hypoxia, TNF release, HIF-1α, TGF-β, and TP53 mutations in the tumor environment [34–38]. Considering HCC is characterized by rich blood supply, hypoxia conditions may be less serious than in many other tumor environments. This difference from other tumors may explain why HCC cells express little CD39, as confirmed in previous studies [26] and our in vitro results.

However, we found HCC cells, though barely expressing CD39 themselves, subjectively elevated CD39 expression on macrophages by exosomal circTMEM181. This spatially isolated CD39–CD73 expression indicates a special niche (CD39^+^ macrophages surrounded by CD73^+^ HCC cells) in the HCC environment. Notably, our results indicate that the formation of these niches may lead to poor response to the PD1 antibody in HCC. In fact, PD1 antibody-mediated tumor cell death in HCC releases the most eATP in the HCC environment apart from cell death due to hypoxia. The isolated ATP–adenosine pathway may exist before anti-PD1 therapy and become enhanced after anti-PD1 therapy. However, whether activated before or after immunotherapy, the cross talk between HCC and macrophages forms an environment that helps HCC to resist anti-PD1 therapy and survive.

## Conclusion

In summary, our research suggests for the first time that HCC cells act on macrophages through exosomal circTMEM181 to increase their CD39 expression. Coordinated with CD73 on tumor cells, this process eventually leads to adenosine elevation in the tumor environment, disabling CD8^+^ T cells and causing resistance to anti-PD1 immunotherapy. CD39 is the key molecule in this process; therefore, targeting CD39 may benefit anti-PD1-resistance in patients with HCC.

## Supplementary Information

**Additional file 1.** Supplementary Figures.

**Additional file 2.** Supplementary Materials.

## Data Availability

Data in this study are included in the main article or in Additional file 2.

## Abbreviations

HCC: Hepatocellular carcinoma
PD1: Programmed cell death 1 protein
PD-L1: Programmed cell death 1 ligand 1
TME: Tumor microenvironment
OS: Overall survival
circRNA: Circular RNA
PR: Partial response
PD: Progressive disease
TMA: Tissue microarray
TIL: Tumor-infiltrating leukocytes
FISH: Fluorescent in situ hybridization
IHC: Immunohistochemistry
eATP: Extracellular ATP
ADO: Adenosine
TAM: Tumor-associated macrophages
sCD73: Soluble CD73
