# Supplementary material for: Amplification of spatially isolated adenosine pathway by tumor–macrophage interaction induces anti-PD1 resistance in hepatocellular carcinoma
Source: J Hematol Oncol. 2021 Nov 27;14:200. doi: 10.1186/s13045-021-01207-x (PMC8627086; doi:10.1186/s13045-021-01207-x)
Supplement: Supplementary file 1 — Additional file 1. Supplementary Figures. [file 13045_2021_1207_MOESM1_ESM.pdf]

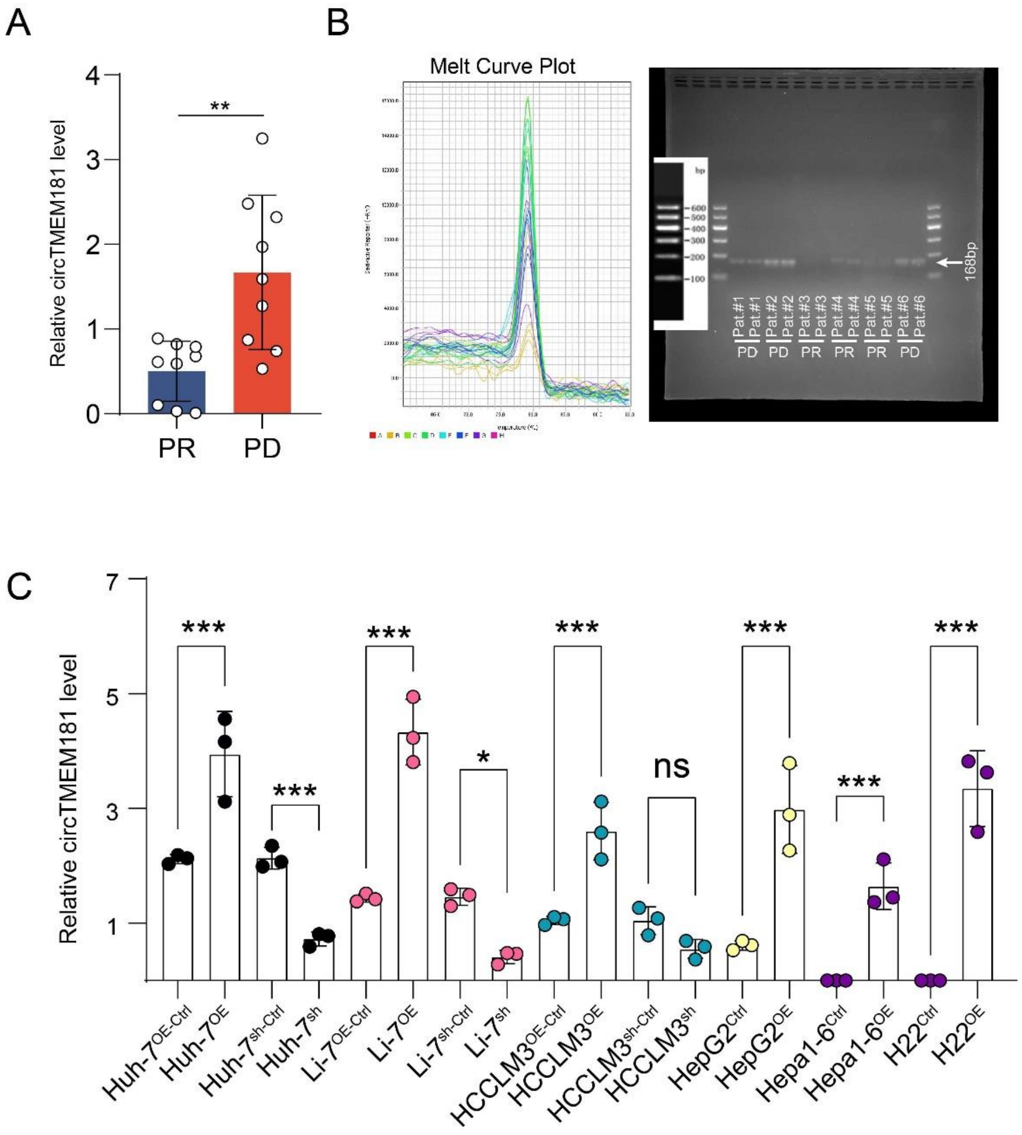

Supplementary Figure 1. Validation of circTMEM181 expression in HCC

(**A**) qPCR analysis validated the circTMEM181 level in 6 patients with PD or PR after anti-PD1 therapy (tests were repeated three times for each patient, t-test, **: $p<0.01$)

(**B**) Melt curve plot and gel electrophoresis validated the amplified PCR product by reverse transcription (RT)-PCR utilizing a divergent primer pair of hsa_circ_0001663(circTMEM181) in HCC tumor (6 Patients with PD or PR after anti-PD1 therapy).

(**C**) qPCR analysis indicated that different HCC cell lines stably overexpressing circTMEM181 (OE) or circTMEM181 knock down (sh) were constructed by lentivirus.

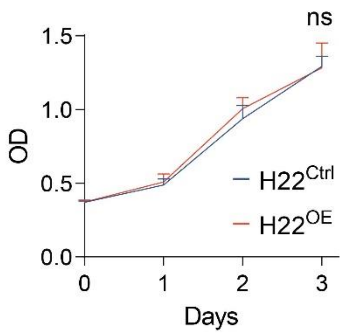

Supplementary Figure 2. Function of circTMEM181 in H22 cells

CCK-8 analysis showed no statistical difference in the proliferative capacity between H22 overexpressing circTMEM181(H22^{OE}) and the negative control (H22^{Ctrl}) at Day 3 (t-test, ns: not significant).

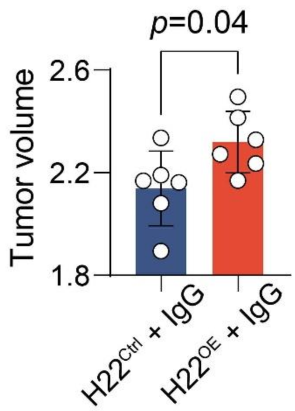

Supplementary Figure 3. Tumor volume of the H22 ^{Ctrl} + IgG and H22 ^{OE} + IgG

H22^{Ctrl} + IgG and H22^{OE} + IgG mice were sacrificed at Day 20 and the tumor volume was measured (t-test).

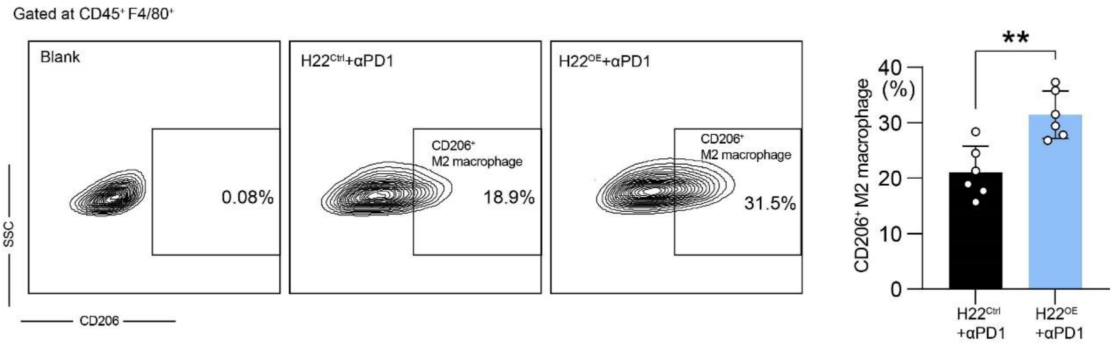

Supplementary Figure 4. Detection of CD206^{+} macrophage between H22^{OE}+αPD1 group and H22^{Ctrl}+αPD1 group

Flow cytometry analysis shows higher proportion of CD206^{+} M2 expression in H22^{OE}+αPD1 group (t-test, **: p<0.01)

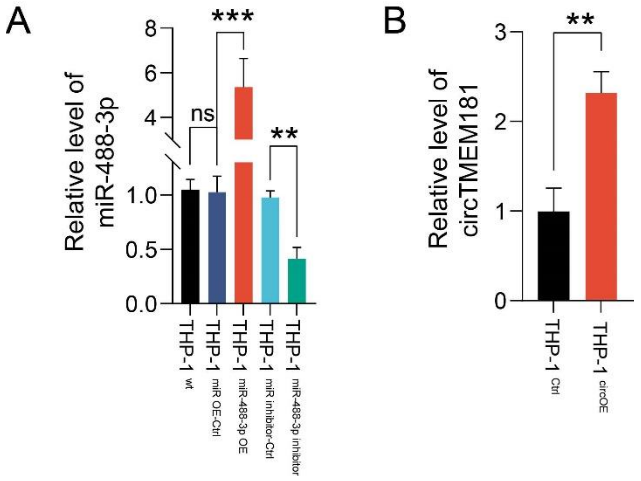

Supplementary Figure 5. Manipulating circTMEM181 and miR-448-3p in THP-1

(**A**) qPCR analysis indicated that THP-1 was stably overexpressed or inhibited miR-488-3p (t-test, **: p<0.01).

(**B**) qPCR analysis indicated that THP-1 was stably overexpressed circTMEM181 (t-test, ***: p<0.001, **: p<0.01, ns: not significant).

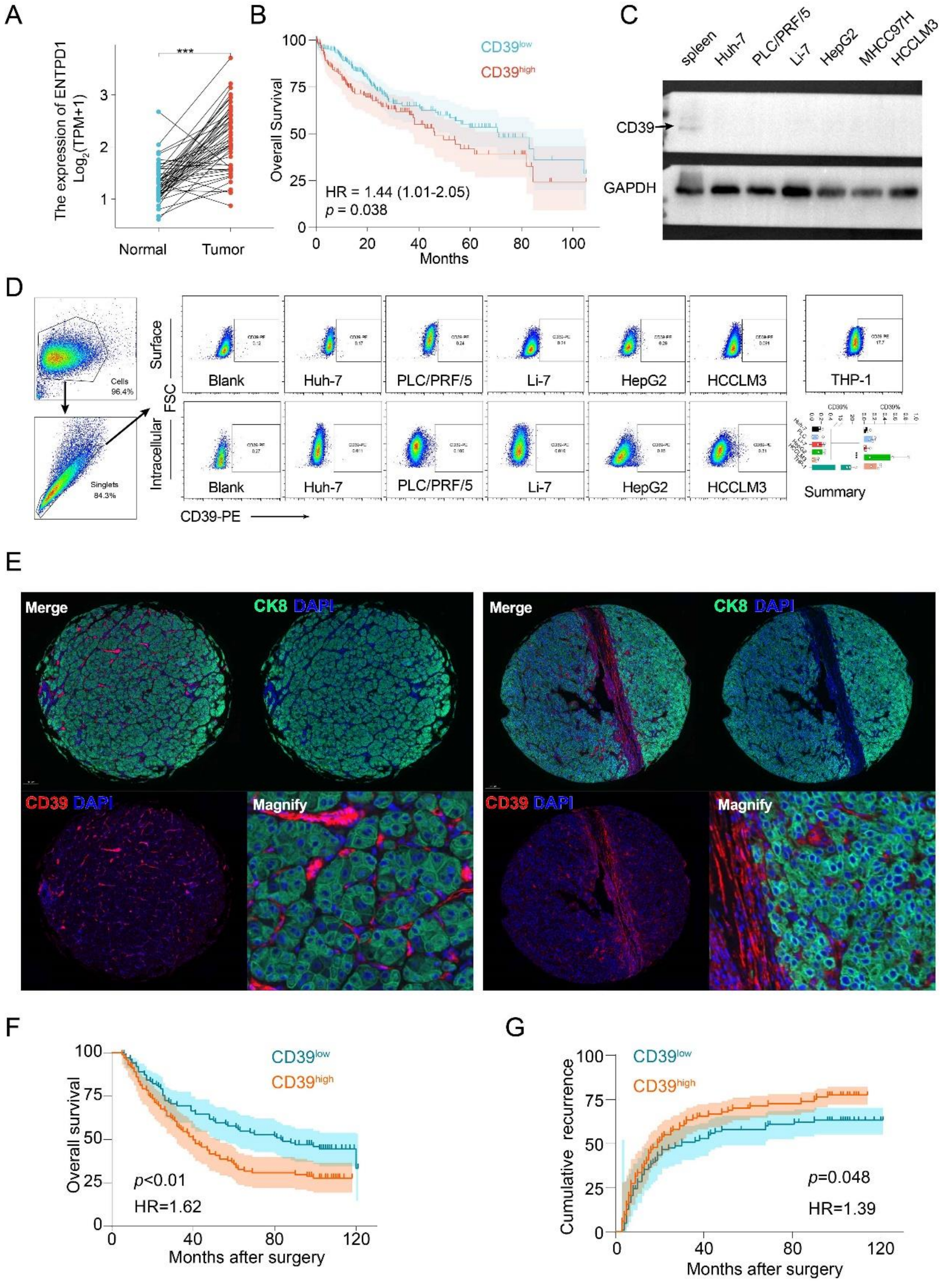

Supplementary Figure 6. Detection of CD39 expression in HCC

(**A**) Analysis of CD39 expression in 50 paired HCC and corresponding normal tissue from TCGA data (paired-t test, ***: p<0.001).

(**B**) Kaplan-Meier estimate of overall survival in the TCGA HCC cohort with different levels of CD39 (log-rank test).

(**C**) WB analysis showed CD39 and GAPDH expression across the HCC cell lines (Huh-7,

PLC/PRF/5, Li-7, HepG2, 97H and HCCLM3) and the positive control (spleen tissue).
(**D**) Flow cytometry analysis of CD39 expression on the HCC cell lines (Surface or Intracellular), THP-1 (t-test, ***: $p<0.001$, ns: not significant).
(**E**) Immunofluorescence showed CD39 expression on HCC TMA (Green: CK8, Blue: DAPI, Red: CD39).
(**F** and **G**) Kaplan-Meier estimate of overall survival or cumulative recurrence in the cohort with different levels of CD39 (n=204, log-rank test).

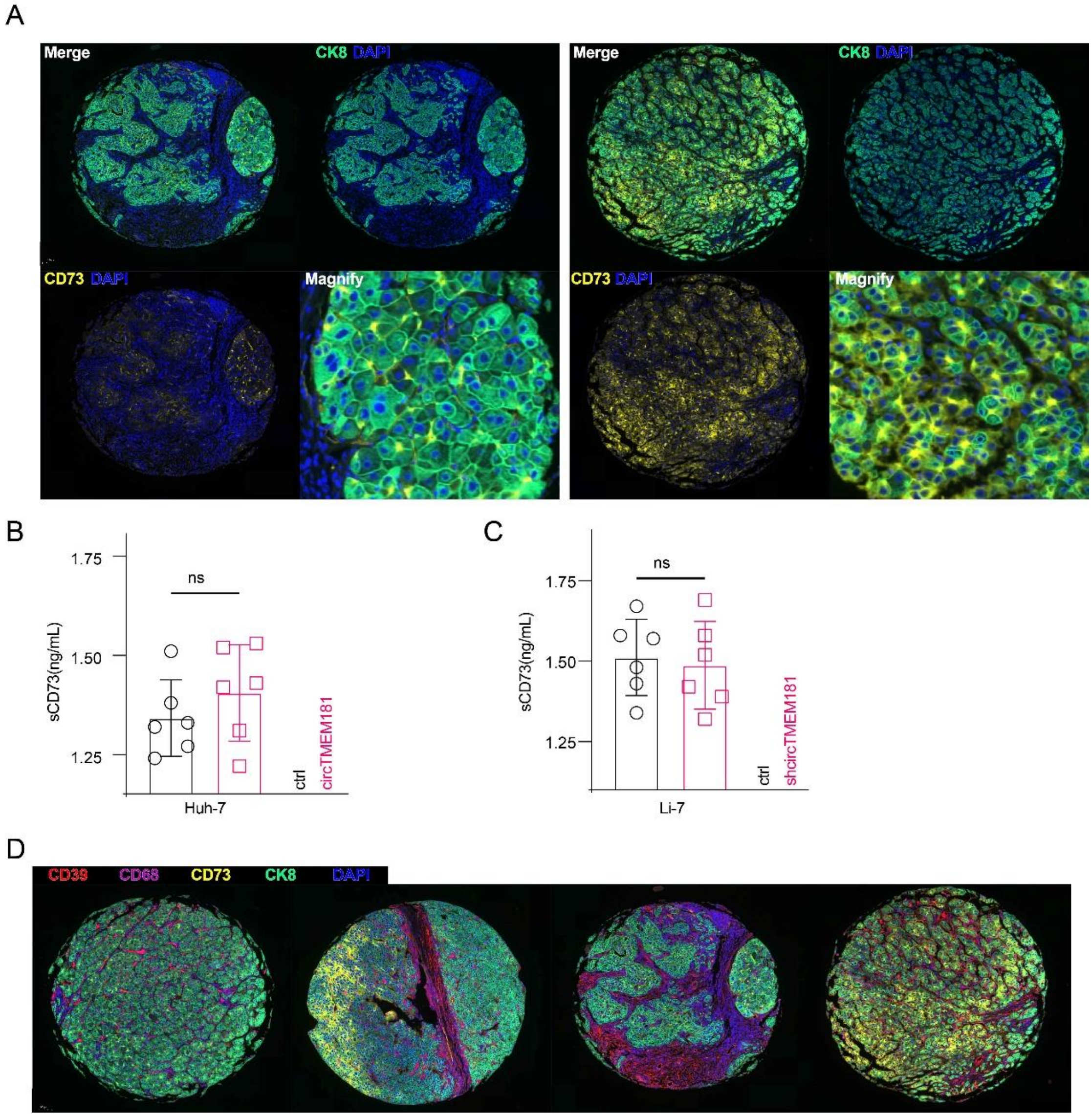

Supplementary Figure 7. Detection of CD73 expression in HCC

(**A**) Immunofluorescence showed CD73 expression on HCC TMA (Green: CK8, Blue: DAPI, Yellow: CD73).

(**B**) Soluble CD73 (sCD73) was detected in indicated conditions (t-test: ns: not significant).

(**C**) Multi-label-immunofluorescence showing the expression of CD39 (Red) on CD68^{+} macrophage (Purple), and the expression of CD73 (Yellow) on CK8^{+} HCC tumor cells (Green) in HCC TMA.

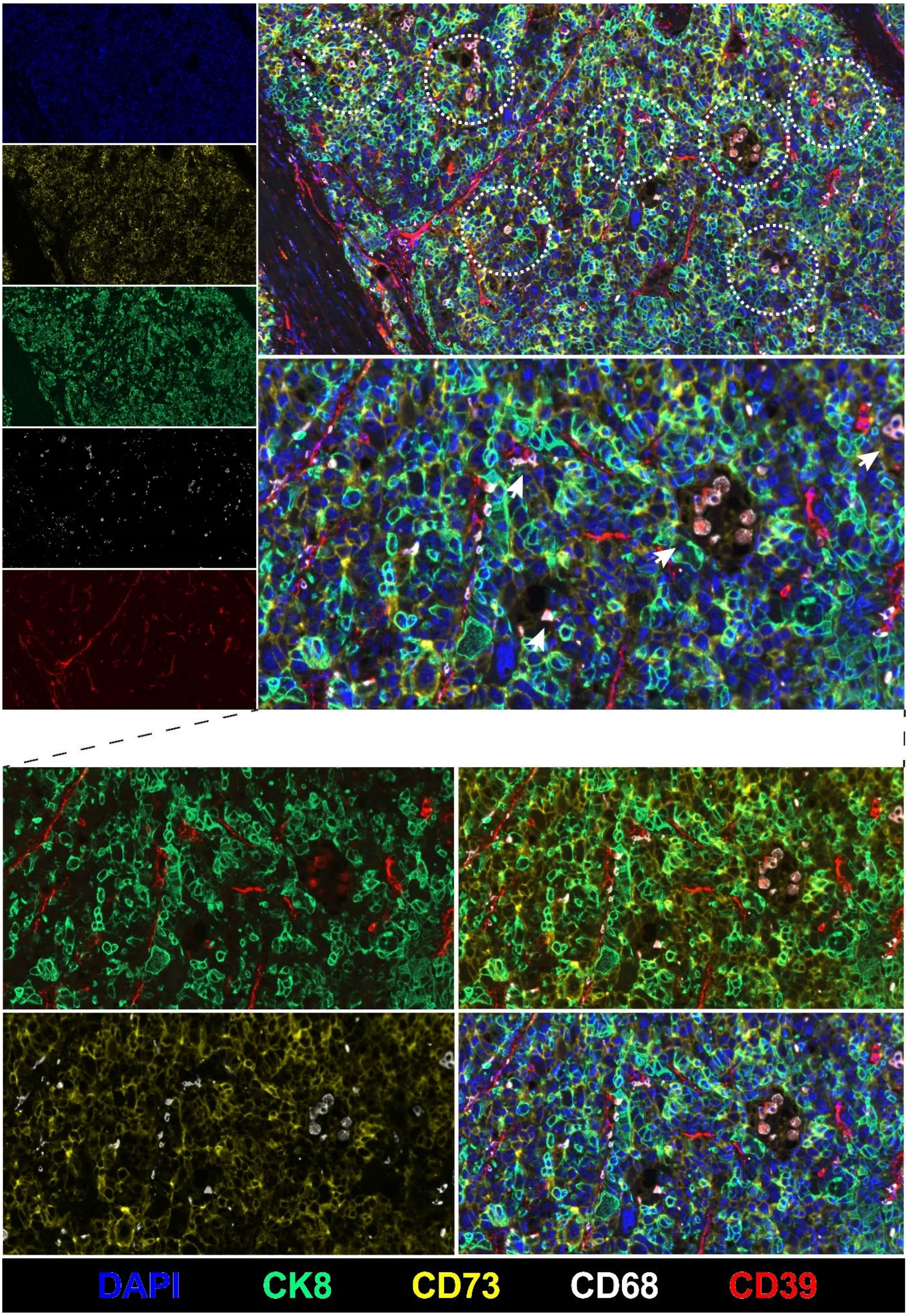

Supplementary Figure 8. High resolution picture for Figure 5H

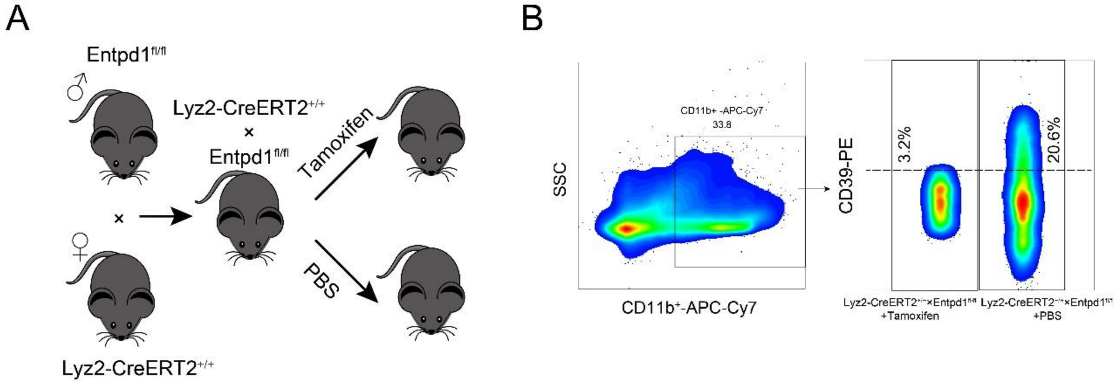

Supplementary Figure 9. Construction of C57-Lyz2CreERT2 × C57 -Entpd1fl/fl mice model

(**A**) Schematic illustration indicated the construction of C57-Lyz2^{CreERT2} × C57 -Entpd1$^{fl/fl}$ mice model.

(**B**) Flow cytometry analysis to verify the selective deletion of CD39 on the macrophage after tamoxifen use.

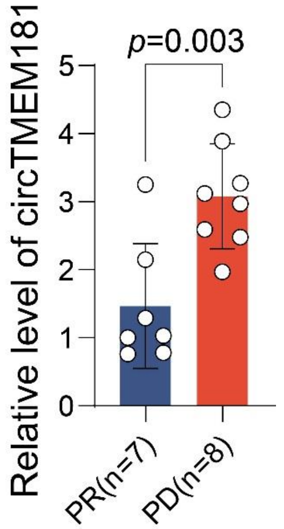

Supplementary Figure 10. Expression of circTMEM181 in the discovery cohort and the validation cohort.

qPCR analysis validated the circTMEM181 level in 15 patients (PD: 8 patients; PR: 7 patients) (t-test).

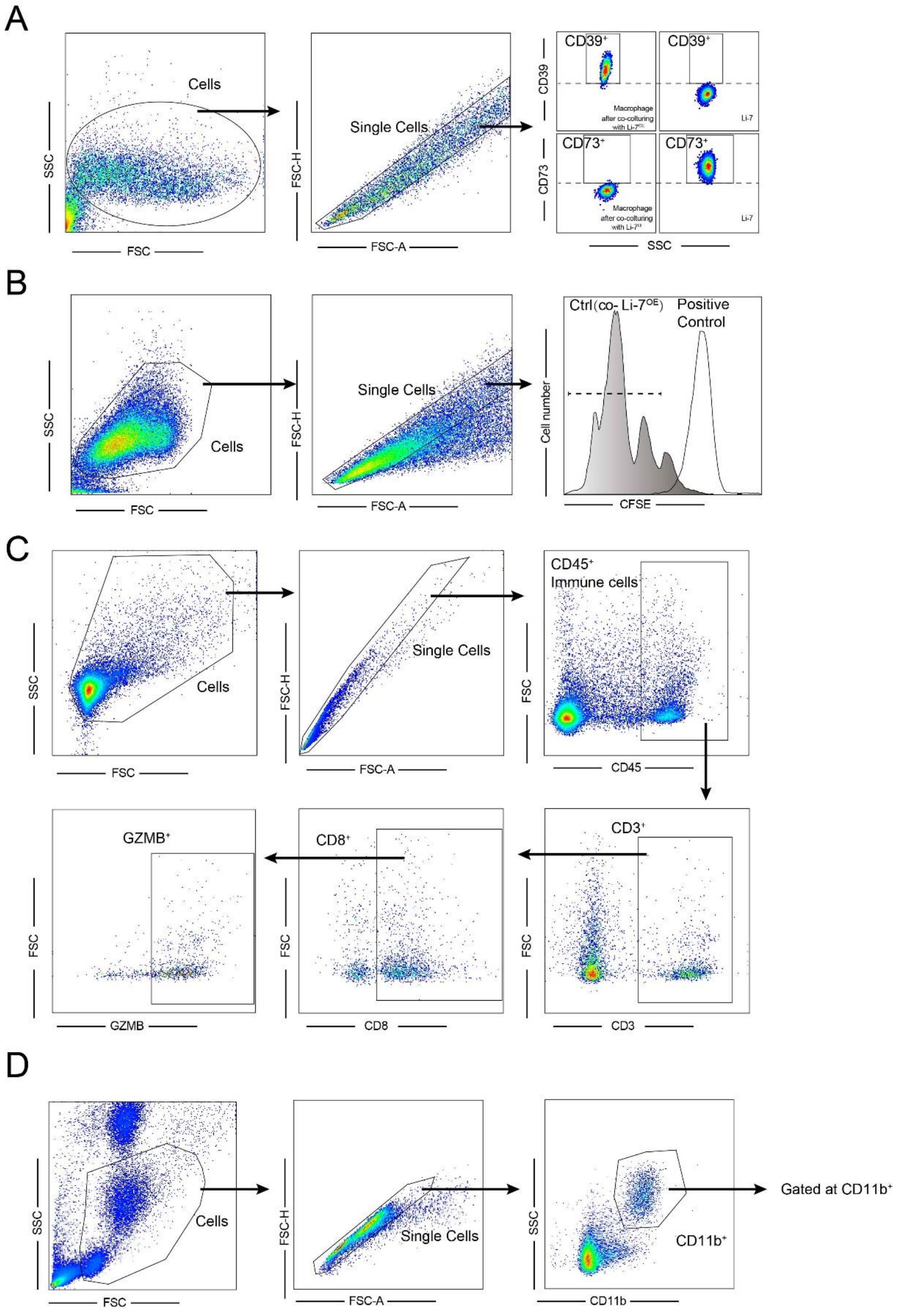

Supplementary Figure 11. Flow cytometry gating strategies

(**A**) Gating strategies for Figure 5B

(**B**) Gating strategies for Figure 5E

(**C**) Gating strategies for Figure 6B, D, J

(**D**) Gating strategies for Figure 7E, H
