# Supplementary material for: Amplification of spatially isolated adenosine pathway by tumor–macrophage interaction induces anti-PD1 resistance in hepatocellular carcinoma
Source: J Hematol Oncol. 2021 Nov 27;14:200. doi: 10.1186/s13045-021-01207-x (PMC8627086; doi:10.1186/s13045-021-01207-x)
Supplement: Supplementary file 2 — Additional file 2. Supplementary Materials. [file 13045_2021_1207_MOESM2_ESM.pdf]

EXPERIMENTAL MODEL AND SUBJECT DETAILS

Tissue microarray and human samples

Tumor tissue samples and/or adjacent nontumorous samples were obtained from HCC patients underwent curative liver resection between 2018 to 2019 at the Liver Cancer Institute, Zhongshan Hospital, Fudan University. Biopsy samples and blood samples were collected from advanced HCC patients before nivolumab therapy. PBMCs were isolated from the healthy donors. Tissue microarray (TMA) were constructed by Shanghai Biochip Co. Ltd. (Shanghai, China), containing the tumor tissues and para-tumors tissue from 204 HCC patients who underwent liver surgery between 2006 to 2008 at the Liver Cancer Institute, Zhongshan Hospital, Fudan University. Tumor differentiation was defined according to the Edmondson grading system. All the HCC tissues were reviewed and confirmed histologically by H&E staining. The collection of human samples was approved by the Zhongshan Hospital Research Ethics Committee. Written informed consent was obtained from each patient.

qPCR

Total RNAs or miRNAs were extracted from cultured cells or fresh-frozen HCC tissue samples with the MolPure® Cell/Tissue Total RNA Kit (Yeasen Biotechnology (Shanghai) Co., Ltd.) or Tissue & Cell microRNA Extraction Kit (HaiGene, Harbin, China) according to the protocols.

Real-time PCR for mRNA or circRNA was performed by using Hieff® qPCR SYBR Green Master Mix (Low Rox Plus, Yeasen Biotechnology (Shanghai) Co., Ltd.). For miRNA, cDNA synthesis was performed using TaqMan miRNA cDNA Synthesis Kit (HaiGene, Harbin, China) and qPCR for miRNA was performed by HG TaqMan miRNA PCR Kit (HaiGene, Harbin, China) according

to the protocols. Targeted miRNAs were normalized to U6B RNA levels using the $2^{-\Delta Ct}$ model. Targeted mRNA or circRNA levels were adjusted using GAPDH. qPCR assays were performed in triplicate for each sample, and the mean value ± SD was used for the statistics of RNA expression levels.

Western blot

Cells or tissues were lysed by RIPA lysis buffer (Yeasen Biotechnology (Shanghai), Co. Ltd.) and the proteins were quantified using the BCA Kit. Proteins were separated on 10 or 12.5 % SDS-PAGE gels, transferred to PVDF membranes (Millipore) and blocked with Protein Free Rapid Blocking Buffer (Epizyme Biotech). After incubating overnight at 4 °C with the primary antibodies described in the Key Resources Table, the PVDF membranes were incubated with HRP-conjugated secondary antibody for 1 h at room temperature. Immersed the PVDF membranes with ECL Detection Reagent (Yeasen Biotechnology). The immunoblots were then detected using a gel image analysis system.

RNA ISH and RNA FISH

Formalin-fixed paraffin-embedded slides were firstly incubated at 70°C for 40 min, then performed deparaffinization following dimethylbenzene and graded alcohol, rinsed with cold tap water for three times. The slides were then boiled in the retrieval solution for 15 minutes. After cooling to room temperature, added proteinase K(20 μg/ml) to cover the tissue and incubated at 37°C for 8 minutes. Washed three times with PBS. Added Pre-hybridization solution to each section and incubated for 1 h at 37°C. Discarded the pre-hybridization solution, and the slides were hybridized

with probes for the targeted nucleic acid overnight at 40°C. Washed the slides with 5 ×, 1 ×, and 0.2 × saline-sodium citrate (SSC) at room temperature.

For FISH, stained cell nuclei by incubating with DAPI for 5 min in the dark, and then mounting with anti-fluorescence quenching sealing tablets. Microscopic examination and photography were then performed.

For ISH, added blocking solution to the section and incubated at room temperature for 30 min. After removing the blocking solution, anti-DIG-HRP was added and incubated at 37 °C for 40 min. Then, washed the sections in TBS four times for 5 min each. Dried sections slightly, and added NBT/BCIP to mark the tissues, finally stopped the reaction by wash in running tap water.

Cell lines and cell culture

All the identified cell lines were provided from Key Laboratory of Carcinogenesis and Cancer Invasion, Ministry of Education of P.R.C or National Collection of Authenticated Cell Cultures, P.R.C. Cell lines HepG2, HCCLM3, MHC97H, Huh-7, PLC/PRF/5, H22, Hepa1-6 were cultured in DMEM with 10% fetal bovine serum. The cell lines THP-1 and Li-7 were cultured in RPMI-1640 with 10% fetal bovine serum. All the cells were cultured at 37 °C in a 5% CO_2 incubator.

Clonogenic assay

Clonogenic assay were performed in six-well plates and seeded cells for each plate. After culturing for 2 weeks, removed the medium above the cells and rinsed carefully with PBS for three times. Then removed the PBS and add 2 ml 4% paraformaldehyde. After waiting for 15 min, removed the 4% paraformaldehyde and rinsed with PBS for three times. Added Crystal Violet Staining Solution

(0.5%) and leaved this for 20 min. Removed the Crystal Violet Staining Solution carefully and rinse with PBS. Leaved the plates to dry in normal air at room temperature for 1 day. Counted the number of colonies under the microscope.

Transwell assays and cell co-culture

For Transwell assay, medium with 10% fetal bovine serum were added to the multiwell plate, followed by adding the Transwell inserts, and lastly adding the cells with medium without fetal bovine serum to the inside compartment. After 3-7 days, the Transwell inserts were removed and rinsed with PBS for three times. Then the Transwell inserts were immersed in 4% paraformaldehyde for 15 min and the cells inside compartment were wiped with a cotton swab. Rinsed with PBS for three times, added Crystal Violet Staining Solution (0.5%) and leaved this for 20 min. Washed the Crystal Violet and counted the number of the cells under the microscope after PBS wash.

For cell co-culture, one of the cells was added to the bottom of the multiwell plate with 10% fetal bovine serum and other needed reagents, followed by adding the Transwell inserts (pore size=0.4 μm), and finally added the second cell with the medium to the inside compartment.

Immunohistochemistry and multiplex immunofluorescence

Formalin-fixed paraffin-embedded slides or TMA slides were incubated at 70°C for 40 min, then performed deparaffinization following dimethylbenzene and graded alcohol (95%, 85%,75%), rinsed with PBS for three times. Antigen retrieval was then performed by EDTA Antigen Retrieval Solution or Citrate Antigen Retrieval Solution at 95°C for 15 min. Rinsed with PBS for three times and blocking endogenous enzymes by Endogenous Peroxidase Blocking Buffer for 10-25 min.

Rinsed with PBS for three times and incubated with Normal Goat Serum For Blocking for 20 min. Removed the blocking solution on the slide, and added the primary antibody working solution to completely cover the sample. Incubated at 4°C overnight. Washed the slides for 3 times with 1× TBST for 2 min each time. After removing the washing solution remaining on the slide, added HRP secondary antibody working solution, and immersed the sample area. Incubated at room temperature for 10 minutes and washed the slides 3 times with 1 × TBST.

Removed the washing solution remaining on the glass slide and added 1 × NEON TSA fluorescent dye to immerse the sample area. Incubated at room temperature for 5 min and washed the slides 3 times with 1 × TBST.

For multiplex immunofluorescence, repeated the steps above since antigen retrieval with different primary antibodies and fluorescent dye. Added anti-fluorescence quenching sealing tablets with DAPI and then mounted the slide with a cover glass. Imaging was performed by Pannoramic MIDI (3DHISTECH Ltd.).

For Immunohistochemistry, after washing the HRP secondary antibody, dried sections slightly, and added DAB to mark the tissue for 10 min. Added Hematoxylin staining solution, staying for 50 sec and washed in tap water. Immersed the slide in water for 30 min. Performed dehydrate following gradient ethanol (75%, 85%, 100%) and dimethylbenzene. Finally, mounted the slide with resin mounting medium and covered glass.

Mice model and *in vivo* tumor models

Male C57BL/6J mice aged 6-week-old were purchased from Vitalriver Laboratory Animal Co., Ltd (Beijing, China). C57BL/6-Entpd1$^{fl/fl}$ (CKOCMP-12495-Entpd1-B6J-VA) and C57BL/6-Entpd1^{KO}

(KOCMP-12495-Entpd1-B6N-VA) were constructed by Cyagen (Suzhou, Jiangsu, China). C57BL/6-Lyz2^{CreERT2} were constructed by Shanghai Model Organisms Center, Inc. (Shanghai, China). C57BL/6-Lyz2^{CreERT2} × C57BL/6-Entpd1$^{fl/fl}$ mice were constructed in our lab. Silencing ENTPD1 in macrophage was activated by treated with tamoxifen (dissolved in corn oil, 20mg/ml by shaking overnight at 37°C) i.p. (100ul/mouse, for 5 days). The mice were housed and bred in SPF facility. All the experiments were approved by the animal welfare board in Zhongshan Hospital, Fudan University.

Subcutaneous tumor xenograft in mice model: 1×10^6 H22 mice liver tumor cells in 0.1 ml of DMEM were implanted subcutaneously in the right flank of the mice. The tumor volume was screened by caliper every three days.

Orthotopic tumor xenograft in mice model: 1×10^6 H22 mice liver tumor cells labeled luciferase (H22-luc) in 50uL Matrigel were implanted to construct orthotopic liver xenografts in the mouse. The tumors were screened by optical *in vivo* Imaging system.

Treatment was performed 7 days later after tumor construction. Anti-mouse PD1 was intraperitoneally injected (10 mg/kg, 3 times every week, for 2 weeks). POM1 was intraperitoneally injected (5 mg/kg, 4 times every week, for 2 weeks). Clophosome was intraperitoneally injected (0.2 µl/mouse, every week, for 2 weeks).

ATP, AMP, ADP and Adenosine measurement

Prepared standard products (ATP, AMP, ADP and adenosine) and dissolved in concentration of 10 µg/mL with ultrapure water. Injected 10 µl to the machine (Agilent 1260 Infinity II) and record the chromatogram. Added 300 µl Extract 1 to 200 µl sample and stayed at 4°C for 1 h. Centrifuged at

4°C, 15, 000 rmp for 10 min and took 200 μl of the supernatant, add an equal volume of Extract 2, mix well, centrifuge at 15,000 rmp for 10 min, take the supernatant, and filter with a 0.22um needle filter. Inject 10ul on the machine and record the chromatogram. The chromatogram acquisition and integration of the compound is performed by the software Chemstation (Agilent).

ELISA

Supernatants were collected from medium, and IL-10 and TNF-α were analyzed using ELISA kits (R&D Systems) according to the instructions.

RNA sequencing

RNA sequencing and bioinformatics analysis were performed by Majorbio (Shanghai, China). In brief, tumor tissue or cell samples were subjected to total RNA extraction with TRIzol reagent. For circRNA sequencing, ribosomal RNAs and linear RNAs were removed by Ribo-Zero Gold rRNA Removal Kit and RNase R to enrich circRNA. Enriched RNAs were amplified and transcribed to create the final cDNA library. Next generation sequencing cDNA libraries was performed on the Illumina PE150 platform.

SILAC (stable isotope labeling by amino acids in cell culture)

SILAC (stable isotope labeling by amino acids in cell culture) was performed according to the protocol (doi: 10.1038/nprot.2006.427). In brief, to investigate different cell protein profiles, THP-$1^{co\text{-}exoOE}$ was cultured in a growth medium with 'heavy' amino acids and THP-$1^{co\text{-}exoCtl}$ was cultured in a growth medium with 'light' amino acids. While a reverse SILAC experiment that switched

heavy and light medium was also performed. The cells were collected and mixed (‘heavy’: ‘light’=1:1), and analyzed with mass spectrometry. Intensity of MS signals between ‘light’ and ‘heavy’ peptides was identified to show the different protein abundance between cells.

Optical in vivo Imaging

Mice were anaesthetized with sodium pentobarbital. Then the mice were administrated D-Luciferin (dissolved in PBS, 150 mg/kg) by intraperitoneal injection. After waiting for 15 min, fluorescence imaging was performed by the imaging system IVIS Lumina K (PerkinElmer) with the software Living Image 4.4.

Exosome isolation

Balanced the sample of medium, cell lysis or serum with PBS, and centrifuged at 1, 500 g, 4°C for 15 minutes. The supernatant then centrifuged at 10, 000 g, 4°C for 30 minutes. Acquired the supernatant after centrifugation and centrifuged for 30 minutes at 14, 000 g, 4°C. After centrifugation, transferred the supernatant to an ultra-high-speed centrifuge tube, and used a pipette to balance with 1×PBS buffer on a balance that can be accurate to 0.001 g, and carefully placed it in the ultra-high-speed centrifuge at 4°C, 110, 000 g ultra-high-speed centrifugation for 120 minutes. After centrifugation, carefully discarded the supernatant, resuspended the pellet in 1 × PBS buffer, and centrifuged at 4°C, 110,000 g, ultra-high speed for 60 minutes. After centrifugation, the supernatant was carefully discarded. The remaining liquid at the bottom of the centrifuge tube was the exosomes. Used 300 μl of 1 × PBS to carefully blow the bottom of the centrifuge tube and suck it into the 0.5-1.5 ml centrifuge tube.

Dual luciferase reporter assay

HEK 293T cells were co-transfected with a luciferase reporter vector and miR-488-3p or miR-1298 mimics or the negative control using Lipo-fectamine 2000 reagent. After 48 h, Firefly and Renilla luciferase signals were then determined by dual-luciferase reporter assay (Promega, USA).

Tumor dissociation

Tumor dissociation were performed following the protocol from Tumor Dissociation Kit, mouse (#130-096-730, Miltenyi Biotec) or Tumor Dissociation Kit, human (#130-095-929, Miltenyi Biotec). In brief, enzymes were prepared by reconstitution of the lyophilized powder, then we prepared enzyme mix by adding the components into a gentleMACS™ C Tube, including DMEM and the enzymes. Tumor tissue from mice or HCC patients was cut into small pieces and transferred into the C Tubes. Then the gentleMACS™ Dissociator were used to perform dissociation program. After termination of the program, the C Tubes containing tumor tissue were detached and incubated at 37 °C under continuous rotation. After incubation for certain time according to the protocol, resuspended sample and applied the cell suspension to a strainer (70 µm) placed on a 50 ml tube. Washed the strainer with 10 ml RPMI 1640 and centrifuged cell suspension at 35 0 × g for 5 min. Aspirated supernatant and performed further experiment.

Flow cytometry and sorting

Isolated single-cell suspensions from the blood or tissue of human or mouse were centrifuged (350g, 5min, at 4°C) in a centrifuge and resuspended in staining buffer (1% FBS in PBS) on ice. Calculated

the cells and transferred 10^6 cells into each staining tube. All the samples were placed on ice throughout the staining procedure and single-cell suspensions were washed with PBS (4°C) for two times and resuspended in 20 µl staining buffer (1% FBS in PBS). FcBlocks (1µl for each tube) were used to incubate with the single-cell suspensions for 15 min before staining.

For surface staining, the antibodies were incubated with cell suspensions for 20 min in dark place at 4°C. For intracellular staining, saponin was used after fixation by 4% paraformaldehyde, and the antibodies were incubated with cell suspensions for 20 min in dark place at 4°C. For nuclear staining, after fixation and permeabilization by buffer set, the antibodies were incubated with cell suspensions for 20 min in dark place at 4°C.

For staining of peripheral blood samples, after staining, red blood cells were lysed for 4 min at room temperature in red blood cell lysing buffer. Cell suspensions were washed with PBS (4°C) for two times and filtered using a strainer (70 µm) before flow cytometry analysis. Data was analyzed by FlowJo 10.6.2 software.

The surface staining for sorting was the same as that for flow cytometry analysis, except that all the procedure was operated in a sterile environment.

Key resource table		
**REAGENT or RESOURSE**	**SOURCE**	**IDENTIFIER**
**Antibodies**		
Anti-Mouse CD45-BV711	BioLegend	103147
Anti-Mouse CD11c-APC	BioLegend	117309
Anti-Mouse CD8a-BV605	BioLegend	100744
Anti-Mouse CD4-BV510	BioLegend	100559
Anti-Mouse F4/80-PE/Cy7	BioLegend	123113
Anti-Mouse CD163-BV421	BioLegend	155309
Anti-Mouse CD19-Percp/Cy5.5	BioLegend	115533
Anti-Mouse NK1.1-PE/Dazzle594	BioLegend	108747
Anti-Mouse CD3e-FITC	BioLegend	100203
Anti-Mouse CD3e-BV510	BD Pharmingen	563024
Anti-Mouse CD39-PE	BioLegend	143803
Anti-Mouse CD39-PE-Cy7	BioLegend	143806
Anti-Mouse CD73-BV605	BioLegend	127215
Anti-Mouse/Human Granzyme B-FITC	BioLegend	515403
Anti-Human CD45-FITC	BioLegend	304006
Anti-Human/Mouse CD11b-APC/Cy7	BioLegend	101226
Anti-Human GZMB-FITC	BioLegend	372206
Anti-Human CD56-FTIC	BD Pharmingen	562794
Anti-Human CD8-PerCP/Cy5.5	BD Pharmingen	565310
Anti-Human CD4-Alexa 700	BD Pharmingen	557922
Anti-Human CD19-BV605	BD Pharmingen	562653
Anti-Human CD163-PE/Cy7	BioLegend	333614
Anti-Human CD141-BV510	BD pharmingen	563298
Anti-Human CD279 (PD-1)-PE	BioLegend	329905
Anti-Human CD366 (Tim-3)-APC	BioLegend	345011
Anti-Human TIGIT (VSTM3)-PE	BioLegend	372703
Anti-Human CD39 PE	BioLegend	328207
Anti-Human CD39 APC	BioLegend	328209
Anti-human CD73 PE	BioLegend	344003
Human Trustain FcX	Biolegend	422301
Trustain FcX(anti-mouse CD16/32)	Biolegend	101319
Anti-CD8 alpha antibody [EPR21769]	Abcam	ab217344
Anti-CD163 antibody [EPR19518]	Abcam	ab182422
F4/80 (D2S9R) XP® Rabbit mAb	Cell Signaling Technology, Inc.	#70076
Anti-Human CD8(SP16)	Gene Tech (Shanghai) Company Limited	GT211202

Anti-Human CD4(EP204)	Gene Tech (Shanghai) Company Limited	GT219102
NCAM1 (CD56) (E7X9M) XP Rabbit mAb	Cell Signaling Technology, Inc.	#99746S
CD19 (D4V4B) XP® Rabbit mAb	Cell Signaling Technology, Inc.	#90176S
CD68 (D4B9C) XP® Rabbit mAb	Cell Signaling Technology, Inc.	#76437S
CD163 (D6U1J) Rabbit mAb	Cell Signaling Technology, Inc.	#93498
Anti-TSG101 antibody [EPR7131(B)]	Abcam	ab133586
Anti-CD63 antibody [EPR5702]	Abcam	ab134045
Anti-CD63 antibody [EPR21151]	Abcam	ab217345
Anti-CD39 antibody [EPR20627]	Abcam	ab223842
Anti-CD39 antibody [EPR3678(2)]	Abcam	ab108248
Anti-ENTPD1 antibody	Sigma-Aldrich	HPA014067
NT5E/CD73 (D7F9A) Rabbit mAb	Cell Signaling Technology, Inc.	#13160
Anti-Human CD68(PG-M1)	Cell Signaling Technology, Inc.	GM087607
Anti-Human Cytokeratin 8&18	Hangzhou Bailing (Biolynx) Biotechnology Co., Ltd.	BX50145
GAPDH (D16H11) XP® Rabbit mAb	Cell Signaling Technology, Inc.	#5174
**Primers for real-time PCR**		
hsa_circ_0001663-F: CATCAAGGGAATGAACTTCACACT		
hsa_circ_0001663-R: TGAGCTACACCATCGACTTTAACA		
ENTPD1-F: CAAGACCAGACAGAAAGGGG		
ENTPD1-R: CTGTCCCACCCAGATCTCC		
**Probes**		
hsa_circ_0001663 probe: 5'-TTGAATTGGCTTTAGTGTGAAGTTCATTCCCT-3'		
miR-488-3p probe:GACCAAGAAATAGCCTTTCAA		

**Drug**		
PD1 antibody (HRP00262-012) provided as gifts by Hengrui Medicine Com., Jiangsu, China)		
**Regent**		
D-Luciferin, Potassium Salt	Yeasen	40902ES02
CFSE Cell Division Tracker Kit	BioLegend	Cat#423801
GW4869	MedChemExpress (MCE)	HY-19363
Clophosome	FormuMax	F70101C-N
PKH67 Green Fluorescent Cell Linker Mini Kit	Sigma-Aldrich	MINI67
Sodium metatungstate(POM1)	MedChemExpress (MCE)	HY-103259
ATP	Solarbio	A9130
ADO	Solarbio	A8240
ADP	Solarbio	A0180
AMP	Solarbio	SA9510
IL10 ELISA kit	R&D Systems	DY217B
TNF-α ELISA kit	R&D Systems	DY210
Tumor Dissociation Kit, mouse	Miltenyi Biotec	130-096-730
Tumor Dissociation Kit, human	Miltenyi Biotec	130-095-929
True-Nuclear™ Transcription Factor Buffer Set	BioLegend	424401
**Software**		
Prism8	Graphpad	https://www.graphpad.com
FlowJo 10.6.2	Flowjo	https://www.flowjo.com
R 3.6.3	The R Foundation	https://www.r-project.org/
SPSS 20.0	IBM	https://www.ibm.com/analytics/spss-statistics-software
**Others**		
BD LSRFortessa™ X-20 Flow Cytometer	BD Biosciences	N/A
MoFlo XDP	Beckman Coulter	N/A
gentleMACS™ Dissociator	Miltenyi Biotec	N/A
Optima XPN-100	Beckman	N/A
JEM-1230	JEOL Ltd.	N/A
IVIS® Lumina K	PerkinElmer	N/A
Pannoramic MIDI	3DHISTECH Ltd.	N/A
